# Massive Mitochondrial Gene Transfer in a Parasitic Flowering Plant Clade

**DOI:** 10.1371/journal.pgen.1003265

**Published:** 2013-02-14

**Authors:** Zhenxiang Xi, Yuguo Wang, Robert K. Bradley, M. Sugumaran, Christopher J. Marx, Joshua S. Rest, Charles C. Davis

**Affiliations:** 1 Department of Organismic and Evolutionary Biology, Harvard University, Cambridge, Massachusetts, United States of America; 2 Ministry of Education Key Laboratory for Biodiversity Science and Ecological Engineering, Institute of Biodiversity Science, Fudan University, Shanghai, China; 3 Department of Ecology and Evolutionary Biology, School of Life Science, Fudan University, Shanghai, China; 4 Computational Biology Program, Public Health Sciences Division, Fred Hutchinson Cancer Research Center, Seattle, Washington, United States of America; 5 Basic Sciences Division, Fred Hutchinson Cancer Research Center, Seattle, Washington, United States of America; 6 Rimba Ilmu Botanic Garden, Institute of Biological Sciences, University of Malaya, Kuala Lumpur, Malaysia; 7 Faculty of Arts and Sciences Center for Systems Biology, Harvard University, Cambridge, Massachusetts, United States of America; 8 Department of Ecology and Evolution, Stony Brook University, Stony Brook, New York, United States of America; University of Michigan, United States of America

## Abstract

Recent studies have suggested that plant genomes have undergone potentially rampant horizontal gene transfer (HGT), especially in the mitochondrial genome. Parasitic plants have provided the strongest evidence of HGT, which appears to be facilitated by the intimate physical association between the parasites and their hosts. A recent phylogenomic study demonstrated that in the holoparasite *Rafflesia cantleyi* (Rafflesiaceae), whose close relatives possess the world's largest flowers, about 2.1% of nuclear gene transcripts were likely acquired from its obligate host. Here, we used next-generation sequencing to obtain the 38 protein-coding and ribosomal RNA genes common to the mitochondrial genomes of angiosperms from *R. cantleyi* and five additional species, including two of its closest relatives and two host species. Strikingly, our phylogenetic analyses conservatively indicate that 24%–41% of these gene sequences show evidence of HGT in Rafflesiaceae, depending on the species. Most of these transgenic sequences possess intact reading frames and are actively transcribed, indicating that they are potentially functional. Additionally, some of these transgenes maintain synteny with their donor and recipient lineages, suggesting that native genes have likely been displaced via homologous recombination. Our study is the first to comprehensively assess the magnitude of HGT in plants involving a genome (i.e., mitochondria) and a species interaction (i.e., parasitism) where it has been hypothesized to be potentially rampant. Our results establish for the first time that, although the magnitude of HGT involving nuclear genes is appreciable in these parasitic plants, HGT involving mitochondrial genes is substantially higher. This may represent a more general pattern for other parasitic plant clades and perhaps more broadly for angiosperms.

## Author Summary

Recent studies have suggested that plant genomes have undergone potentially rampant horizontal gene transfer (HGT), especially in the mitochondrial genome. Here, using phylogenomic approaches, we demonstrate that as much as ∼40% of the mitochondrial genes in the parasitic plant species Rafflesiaceae are acquired from their hosts via HGT. These transgenes are likely functional in their recipient species and in some cases appear to have displaced native copies in the same genomic location. These results establish for the first time that, although the magnitude of HGT involving nuclear genes is appreciable in parasitic plants, HGT involving mitochondrial genes is substantially higher.

## Introduction

**Figure 1:**
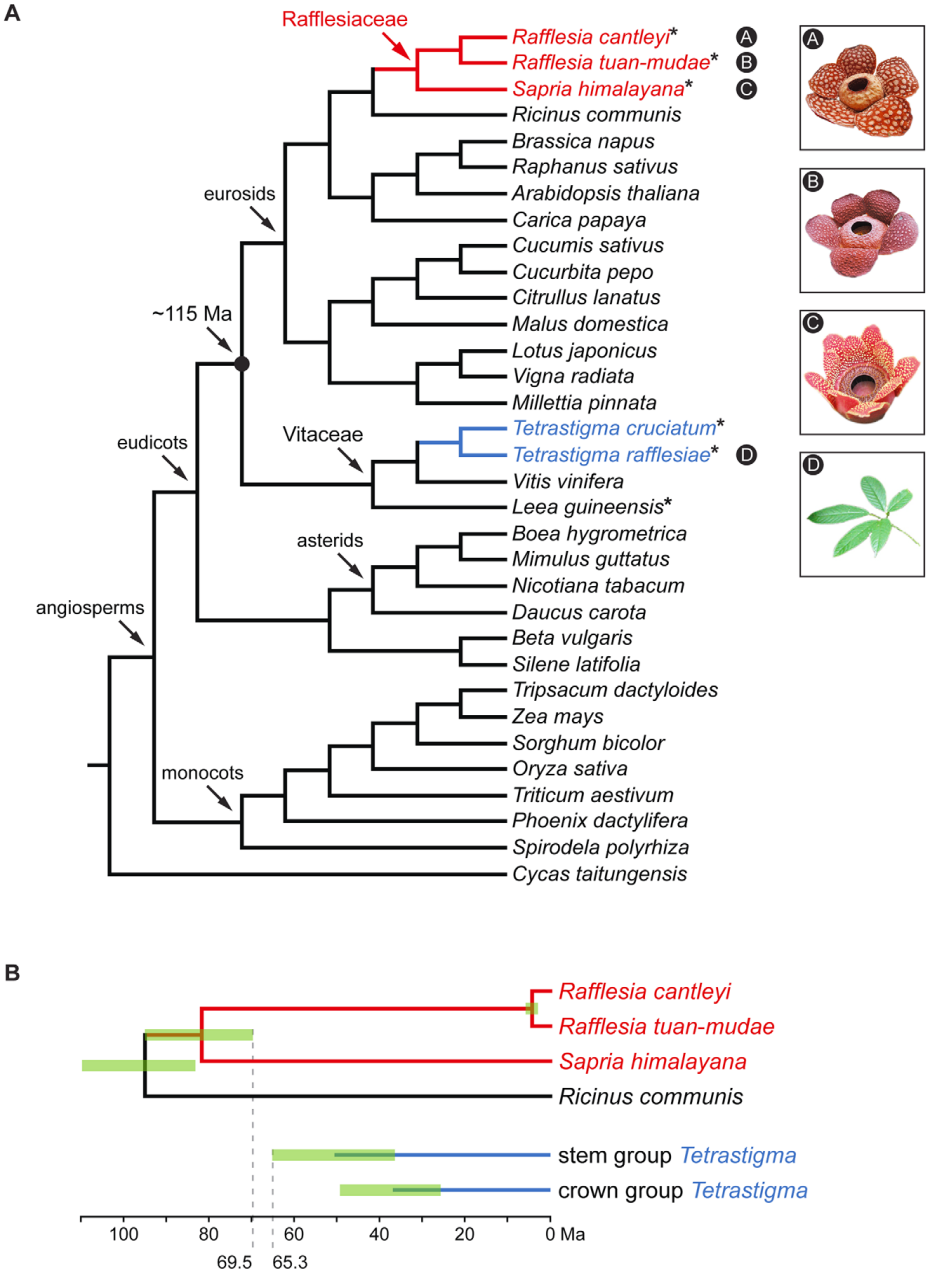
Phylogenetic relationships and divergence times of the three Rafflesiaceae species and two *Tetrastigma* species included in this study. Phylogenetic relationships (A) and divergence times (B). Holoparasitic Rafflesiaceae (red) is a member of the order Malpighiales, and its obligate host *Tetrastigma* (blue) is a member of the Vitaceae family. The approximate divergence time between the parasite and host clade is 115 Ma [24]–[27]. Mitochondrial genome sequences generated in this study are marked with asterisks, and the node age error bars (95% highest posterior density intervals) are shown in green. The accepted phylogenetic relationships are based on APG III [38] and Qiu et al. [40], and the divergence times of Rafflesiaceae and *Tetrastigma* are based on Bendiksby et al. [48] and Chen et al. [49], respectively.

Recent studies have suggested that plant genomes have undergone potentially rampant horizontal gene transfer (HGT) [1], [2], especially in the mitochondrial genome [3]–[7]. Parasitic plants have provided the strongest evidence of HGT [8]–[12], which appears to be facilitated by the intimate physical association between the parasites and their hosts [8], [10], [12]–[15]. One parasitic plant clade that appears to be prone to HGT is Rafflesiaceae *sensu stricto*, which belong to the order Malpighiales [8], [16]–[18] and whose members possess the largest flowers in the world. Rafflesiaceae are endophytic holoparasites, which lack leaves and stems. This family includes the genera *Rafflesia* (∼28 species), *Rhizanthes* (four species), and *Sapria* (three species), and provides one of the best opportunities to investigate HGT in plants because (i) the parasites have a very narrow host specialization range on members of the grapevine family (*Tetrastigma* spp., Vitaceae), (ii) complete genome sequences, including fully annotated mitochondrial and plastid genomes, are available for close relatives of the parasites (*Ricinus communis*, Euphorbiaceae) [19], [20] and their hosts (*Vitis vinifera*, Vitaceae) [21]–[23], and (iii) the hosts and parasites are separated by at least 115 million years of evolution (Figure 1A) [24]–[27]. These factors make it easier to distinguish transgenes from native genes in Rafflesiaceae using phylogenomic tools.

A recent phylogenomic study demonstrated that in *Rafflesia cantleyi*, about 2.1% of nuclear gene transcripts were likely acquired from its obligate host [28]. This study, however, did not include a thorough investigation of the mitochondrial genome. Here, we comprehensively sequenced 38 mitochondrial genes from *R. cantleyi* and five additional species, including two of its closest relatives and two host species. Our results reveal an extraordinarily high degree of HGT in the mitochondrial genome of Rafflesiaceae involving genes that were likely acquired from its host at various time intervals. Most of these transgenic sequences possess intact reading frames and are actively transcribed indicating that they are potentially functional. Additionally, some of these transgenes maintain synteny with their donor and recipient lineages suggesting that native genes in Rafflesiaceae have likely been displaced via homologous recombination. These results establish for the first time that although the magnitude of HGT involving nuclear genes is appreciable in these parasitic plants, HGT involving mitochondrial genes is substantially higher.

## Results/Discussion

### The mitochondrial provenance of our sequenced genes

We used next-generation sequencing to comprehensively sequence the mitochondrial genomes of three species that span the crown node of Rafflesiaceae: *Rafflesia cantleyi*, *Rafflesia tuan-mudae*, and *Sapria himalayana* (Figure 1A, see also Table S1). We then extracted the 38 mitochondrial genes from our *de novo* assembled contigs that ranged in size from 2 to 54 kilobases (kb). These 38 protein-coding and ribosomal RNA genes are present in the mitochondrial genomes of both *Ricinus* and *Vitis*, and are also common to most angiosperms [29]. We included 35, 33, and 59 gene sequences from *R. cantleyi*, *R. tuan-mudae* and *S. himalayana*, respectively, for further analyses. While repetitive sequences made assembly of the entire chromosome impractical, high sequence coverage (Table S1) ensured that we have sequenced all coding regions in these mitochondrial genomes.

Several lines of evidence suggest that all gene sequences we assembled here are localized to the mitochondrial genome of Rafflesiaceae. First, the genome libraries for two of our three Rafflesiaceae species (i.e., *R. cantleyi* and *S. himalayana*) were prepared from fresh tissue using sucrose gradient centrifugation, which are enriched for plant organelles [30]. Since the plastid genome has apparently been lost in Rafflesiaceae [31], our libraries are heavily enriched for mitochondria. Second, plastid, mitochondrial and nuclear genes in plant cells differ widely in copy number: plastid genes are generally present in hundreds to thousands of copies per cell, mitochondrial genes in tens to hundreds of copies per cell, while nuclear genes are usually present in only two copies per cell [7], [32]. To investigate if gene sequences assembled here have copy numbers that correspond with a mitochondrial localization, we compared gene copy number here to 1,305 genes previously determined to be localized to the nuclear genome of *R. cantleyi* [28] and *R. tuan-mudae* [18]. Our results demonstrate that copy numbers for all putative mitochondrial gene sequences in Rafflesiaceae are one to two orders of magnitude greater than for nuclear genes (Table S2), with means of 155-, 68-, and 160-fold greater for *R. cantleyi*, *R. tuan-mudae*, and *S. himalayana*, respectively (p-value<2.2×10^−16^, Welch's *t* test). These copy numbers are consistent with a mitochondrial localization, but not high enough to suggest localization in, or the existence of, a plastid genome in Rafflesiaceae. Third, when comparing assembled gene sequences from *R. cantleyi* with our previously published complementary DNA (cDNA) library [28], we identified cytosine-to-uracil (C-to-U) RNA editing in seven genes (i.e., *atp1*, *atp4*, *atp6*, *cox2*, *nad1*, *rps4*, and *rps12*), which is a common characteristic of mitochondrial genes [33]. These results collectively indicate that these gene sequences are most likely localized to the mitochondrial genome of Rafflesiaceae, although complete assembly of these mitochondrial genomes will be required to definitively confirm our results.

### Extraordinarily high, and variable, rates of HGT in the mitochondrial genome of Rafflesiaceae

To estimate the magnitude of HGT in the mitochondrial genome of Rafflesiaceae, we also sequenced the same 38 mitochondrial genes from three species of Vitaceae: *Tetrastigma cruciatum*, which is the host of *S. himalayana* [34], *Tetrastigma rafflesiae*, which is the host of *R. cantleyi* and *R. tuan-mudae* [34], [35], and *Leea guineensis* (Figure 1A, see also Table S1). The latter represents the earliest diverging lineage of Vitaceae [36], [37], which allows us to determine if putative transgenic sequences from Rafflesiaceae are phylogenetically nested within the host clade Vitaceae.

Our newly sequenced mitochondrial gene sequences from the six species were then analyzed using maximum likelihood (ML) with homologous sequences from 27 other seed plants whose mitochondrial genomes have been sequenced and fully annotated (Figure 1A, see also Table S3; *Arabidopsis thaliana* [Brassicaceae], *Beta vulgaris* [Amaranthaceae], *Boea hygrometrica* [Gesneriaceae], *Brassica napus* [Brassicaceae], *Carica papaya* [Caricaceae], *Citrullus lanatus* [Cucurbitaceae], *Cucumis sativus* [Cucurbitaceae], *Cucurbita pepo* [Cucurbitaceae], *Cycas taitungensis* [Cycadaceae], *Daucus carota* [Apiaceae], *Lotus japonicus* [Fabaceae], *Malus domestica* [Rosaceae], *Millettia pinnata* [Fabaceae], *Mimulus guttatus* [Phrymaceae], *Nicotiana tabacum* [Solanaceae], *Oryza sativa* [Poaceae], *Phoenix dactylifera* [Arecaceae], *Raphanus sativus* [Brassicaceae], *Ricinus communis*, *Silene latifolia* [Caryophyllaceae], *Sorghum bicolor* [Poaceae], *Spirodela polyrrhiza* [Araceae], *Tripsacum dactyloides* [Poaceae], *Triticum aestivum* [Poaceae], *Vigna radiata* [Fabaceae], *Vitis vinifera*, and *Zea mays* [Poaceae]). These reference species represent a broad sampling of most major flowering plant clades [38]. Each Rafflesiaceae gene sequence was placed into one of three categories–i.e., VGT, HGT, or unassigned–on the basis of its phylogenetic position and ML bootstrap percentage (BP) support following Xi et al. [28]. We applied two BP thresholds to categorize each gene sequence. Our more conservative estimate applied a 70 BP threshold; this BP threshold has been shown to correspond to a very high probability that the clade is real [39]. Here, gene sequences whose placements were consistent with accepted species' relationships (i.e., Rafflesiaceae gene sequences were sister to their closest relative *Ricinus* with ≥70 BP; [17], [18]) were scored as VGT; HGT was inferred when gene sequences were placed elsewhere with ≥70 BP; and gene sequences with <70 BP were left unassigned. To explore if our estimates of HGT were sensitive to our thresholds, we also categorized these gene sequences by applying a less conservative threshold using ≥50 BP.

Our phylogenetic analyses of the 38 mitochondrial genes indicated that, for the 30 autotrophic species included here (i.e., 27 reference species, two *Tetrastigma* species, and *Leea*; Figure 1A), phylogenetic placements largely agreed with accepted relationships between families [38], [40] using both the 70 and 50 BP thresholds (Figures S1A and S2). The only three exceptions were for *atp1* where Brassicaceae (i.e., *Arabidopsis*+*Brassica*+*Raphanus*) was placed sister to the asterids (i.e., *Boea*+*Mimulus*+*Nicotiana*) with 81 BP (a similar topology was also identified by Nickrent et al. [10]); for *atp4* where Brassicaceae was placed sister to Fabaceae (i.e., *Lotus*+*Millettia*+*Vigna*) with 93 BP; and for *cox1* where Brassicaceae was placed sister to Caryophyllales (i.e., *Beta*+*Silene*) with 87 BP (Figure S1A). These results indicate that applying both the 70 and 50 BP thresholds yields very low false positive estimates of HGT in these autotrophic species, for which we expect little or no HGT to occur.

**Figure 2:**
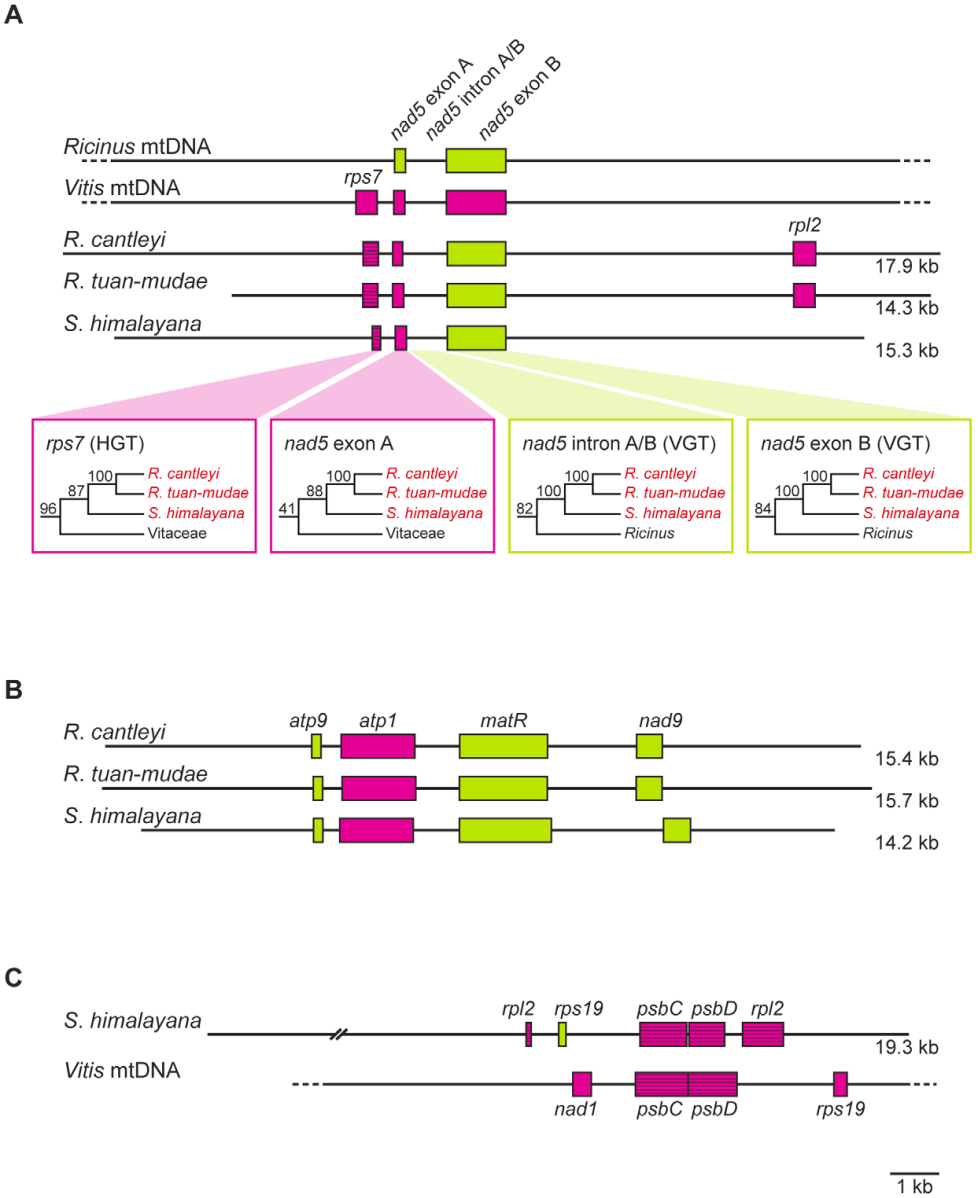
Gene organization of three assembled contigs for Rafflesiaceae (*Rafflesia cantleyi*, *Rafflesia tuan-mudae*, and *Sapria himalayana*), *Ricinus communis* (Euphorbiaceae), and *Vitis vinifera* (Vitaceae). (A–C) The green and red boxes indicate *Ricinus*-like and *Vitis*-like genes, respectively. Pseudogenes are represented by striped boxes, and the sequence length (in kilobases [kb]) is indicated to the right of each assembled contig. Gene organization of *Ricinus* and *Vitis* mitochondrial genomes (mtDNA) follows Rivarola et al. [20] and Goremykin et al. [23], respectively.

In contrast, in the three holoparasitic Rafflesiaceae species, 11 mitochondrial genes demonstrated evidence for one or more cases of HGT using our more conservative 70 BP threshold: of the 21 gene sequences with ≥70 BP in *R. cantleyi*, five gene sequences (24%) showed evidence of HGT, 5 of 19 (26%) in *R. tuan-mudae*, and 11 of 27 (41%) in *S. himalayana*. Furthermore, vertical placements of these putative transgenic sequences were rejected in 18 of 21 cases using the approximately unbiased (AU) test (Table 1). For the less conservative 50 BP threshold, the number of mitochondrial genes that showed evidence of HGT increased to 16; however, the relative frequencies of HGT are nearly identical with those above: 29% in *R. cantleyi* (7 of 24), 32% in *R. tuan-mudae* (7 of 22), and 47% in *S. himalayana* (16 of 34). This indicates that our less conservative threshold does not increase false positive rates. Thus, given the consistency of our estimates of HGT using both thresholds, we treat these transgenes collectively in the discussion below unless otherwise indicated. Two additional findings support the reliability of our HGT inferences: first, the phylogenetic placements of these transgenic sequences were not obviously biased by C-to-U RNA editing (see Figure S1B for phylograms with RNA editing sites excluded from our alignments); second, seven of our large assembled contigs contained both transgenes and native genes (Figure 2), indicating that these transgenes were clearly integrated into the mitochondrial genome of Rafflesiaceae. Therefore, rates of mitochondrial HGT in Rafflesiaceae appear to be extraordinarily high, and well above the false positive rates established from the 30 autotrophic species included here.

**Table 1:** Horizontally transferred (HGT) sequences identified in the mitochondrial genomes of *Rafflesia cantleyi*, *Rafflesia tuan-mudae*, and *Sapria himalayana* with associated statistics.

Gene	Species	Gene identity	HGT donor lineage	BP	AU test	Gene length (base pairs)	Reading frame	RPKM	C-to-U RNA editing site
**Mitochondrial genes**									
***atp1***	*R. cantleyi*	HGT	Vitaceae	54	NS	1520	I	2370	1039
	*R. tuan-mudae*	HGT	Vitaceae	54	NS	1520	I		
	*S. himalayana*	HGT	Vitaceae	54	NS	1514	I		
***atp4***	*R. cantleyi*	HGT	Cucurbitaceae	83	NS	526	I	6560	168, 337
	*R. tuan-mudae*	HGT	Cucurbitaceae	83	NS	526	I		
	*S. himalayana*	HGT	Cucurbitaceae	83	NS	520	I		
***atp9***	*R. cantleyi*	VGT	–	94	–	204	I	1225	
	*R. tuan-mudae*	VGT	–	94	–	204	I		
	*S. himalayana*	VGT	–	94	–	204	I		
	*S. himalayana*	HGT	Vitaceae	65	NS	195	I		
***cob***	*R. cantleyi*	VGT	–	96	–	1166	I	941	
	*R. tuan-mudae*	VGT	–	96	–	1166	I		
	*S. himalayana*	VGT	–	96	–	1166	I		
	*S. himalayana*	HGT	Vitaceae	99	0.0004	1179	I		
***cox1***	*R. cantleyi*	HGT	Brassicaceae+Caryophyllales	71	0.0360	1542	I	449	
	*R. tuan-mudae*	HGT	Brassicaceae+Caryophyllales	71	0.0360	1542	I		
	*S. himalayana*	HGT	Brassicaceae+Caryophyllales	71	0.0360	1542	I		
	*S. himalayana*	HGT	*Tetrastigma*	100	<0.0001	1461	I		
***cox2***	*R. cantleyi*	–	–	<50	–	770	I	2183	14, 61, 153, 233
	*R. tuan-mudae*	–	–	<50	–	770	I		
	*S. himalayana*	–	–	<50	–	771	I		
	*S. himalayana*	HGT	*Tetrastigma*	96	<0.0001	246	I		
***cox3***	*R. cantleyi*	VGT	–	78	–	798	I	1810	
	*S. himalayana*	VGT	–	78	–	798	I		
	*S. himalayana*	HGT	*Tetrastigma*	85	0.0063	337	I		
***rpl2***	*R. cantleyi*	HGT	Vitaceae	51	NS	408	I	44	
	*R. tuan-mudae*	HGT	Vitaceae	51	NS	408	I		
	*S. himalayana*	HGT	*Tetrastigma*	69	NS	963	ψ		
***rpl5***	*R. cantleyi*	VGT	–	89	–	375	I	1619	
	*R. tuan-mudae*	VGT	–	89	–	375	I		
	*S. himalayana*	VGT	–	89	–	528	I		
	*S. himalayana*	HGT	*Tetrastigma*	100	0. 0030	543	I		
***rps1***	*R. cantleyi*	HGT	*Tetrastigma*	100	<0.0001	342	I	155	
	*R. tuan-mudae*	HGT	*Tetrastigma*	100	<0.0001	342	I		
	*S. himalayana*	HGT	*Tetrastigma*	100	<0.0001	494	I		
***rps4***	*R. cantleyi*	VGT	–	96	–	838	I	2358	50, 61
	*R. tuan-mudae*	VGT	–	96	–	838	I		
	*S. himalayana*	VGT	–	96	–	1040	I		
	*R. cantleyi*	HGT	*Tetrastigma*	81	0.0075	143	I	99	
	*R. tuan-mudae*	HGT	*Tetrastigma*	81	0.0075	143	I		
***rps7***	*R. cantleyi*	HGT	Vitaceae	96	0.0060	328	ψ	850	
	*R. tuan-mudae*	HGT	Vitaceae	96	0.0060	328	ψ		
	*S. himalayana*	HGT	Vitaceae	96	0.0060	186	ψ		
***rps13***	*S. himalayana*	HGT	Vitaceae	86	0.0033	264	ψ		
***rps14***	*S. himalayana*	HGT	*Tetrastigma*	68	0.0104	303	I		
***sdh3***	*S. himalayana*	HGT	Vitaceae	96	0.0102	272	I		
***sdh4***	*R. cantleyi*	–	–	<50	–	390	I	2902	
	*S. himalayana*	–	–	<50	–	390	I		
	*S. himalayana*	HGT	Vitaceae	63	NS	243	I		
**Genes of plastid origin**									
***atpA***	*R. tuan-mudae*	HGT	*Daucus*	94	0.0044	515	ψ		
	*S. himalayana*	HGT	*Daucus*	94	0.0044	457	ψ		
***atpB***	*S. himalayana*	HGT	*Tetrastigma*	100	0.0001	477	ψ		
***atpI***	*S. himalayana*	HGT	*Tetrastigma*	94	0.0003	100	I		
***ndhB***	*S. himalayana*	HGT	*Tetrastigma*	86	0.0133	447	I		
***psaB***	*S. himalayana*	HGT	*Tetrastigma*	92	0.0111	362	ψ		
***psbA***	*S. himalayana*	HGT	*Tetrastigma*	100	0.0001	395	ψ		
***psbC***	*S. himalayana*	HGT	Vitaceae	100	0.0004	969	ψ		
***psbD***	*S. himalayana*	HGT	*Tetrastigma*	100	<0.0001	753	ψ		
***rbcL***	*S. himalayana*	HGT	Vitaceae	82	NS	436	ψ		
***rpoC1***	*S. himalayana*	HGT	*Tetrastigma*	100	<0.0001	1613	ψ		
***rpoC2***	*S. himalayana*	HGT	*Tetrastigma*	100	0.0003	2000	ψ		
***rps12***	*S. himalayana*	HGT	Vitaceae	75	0.0304	222	ψ		
***rrn16***	*S. himalayana*	HGT	*Tetrastigma*	87	0.0297	1080	–		
***rrn23***	*S. himalayana*	HGT	*Tetrastigma*	99	0.0002	1638	–		

Vertically transferred (VGT) sequences are additionally listed when they are present. HGT donor lineages are inferred from our maximum likelihood (ML) phylogenies, which are summarized here with ML bootstrap percentages (BP) from Figure S1A and S1D. AU = approximately unbiased test; I = intact reading frame; NS = not significant (i.e., p-value>0.05); ψ = presence of nonsense mutation. Gene expression level reported here is normalized to reads per kilobase per million reads (RPKM) for *R. cantleyi*.

Of the 11 mitochondrial genes that showed evidence of HGT using our more conservative threshold, four (i.e., *cob*, *cox3*, *rpl5*, and *rps4*) maintained both horizontally and vertically transferred homologs, and seven included only transgenic sequences (i.e., *atp4*, *cox1*, *cox2*, *rps1*, *rps7*, *rps13*, and *sdh3*) (Table 1). An additional five mitochondrial genes showed evidence of HGT using our less conservative threshold, one of them (*atp9*) maintained both horizontally and vertically transferred homologs, and four included only transgenic sequences (i.e., *atp1*, *rpl2*, *rps14*, and *sdh4*) (Table 1). Of those genes that included only transgenic copies in Rafflesiaceae all had homologs present in the mitochondrial genome of *Ricinus*, which suggests that they were likely present as native copies ancestrally in Rafflesiaceae and were subsequently displaced by transgenic homologs. One example is illustrated by our assembled contig containing the genes *nad5* exons A and B and *rps7* (Figure 2A). In Rafflesiaceae, *nad5* exon B was identified as a native sequence (84 BP) in our phylogenetic analyses, while *rps7* was identified as a transgene (96 BP). The phylogenetic placement of *nad5* exon A within Vitaceae is also consistent with HGT, but support for this placement is <50 BP (Figure 2A, see also Figure S1C). However, the synteny of *nad5* exons A and B is conserved among *Ricinus*, *Vitis*, and all three Rafflesiaceae species, suggesting that the native copy of *nad5* exon A in Rafflesiaceae may have been displaced by a horizontally transferred DNA fragment via homologous recombination [41]. This hypothesis is further supported by the fact that *nad5* exon A is immediately adjacent to the well-placed transgene *rps7* in Rafflesiaceae, which exactly matches the synteny of *Vitis* but not *Ricinus*. To better locate the recombination breakpoint, we analyzed the intron region between *nad5* exons A and B. This ∼1-kb region is highly conserved across angiosperms and can be easily aligned for phylogenetic analysis. We found that *nad5* intron A/B was clearly identified as a native sequence (82 BP; Figure 2A, see also Figure S1C), therefore, the breakpoint is likely very close to the junction of *nad5* exon A and intron A/B. Although the integration of foreign DNA via homologous recombination is common in bacteria [41], reports of this phenomenon are rare for plants (e.g., *atp1* gene [42] and *rps11* gene [1], [4]). Such direct homologous recombination, which is likely facilitated by the intimate physical association between Rafflesiaceae and their hosts [14], [15] combined with the frequent fusion of plant mitochondria [1], [43]–[45], may obviate the need to invoke a transposable element, bacterium, or virus for catalyzing the insertion of a DNA fragment from donor to recipient in plants.

Additionally, in the mitochondrial genome of *S. himalayana*, we found evidence of HGT involving 14 genes that were potentially of plastid origin using our more conservative threshold, only one of which was also identified in *R. tuan-mudae* (Table 1, see also Figure S1D). Thirteen of these genes support the conclusion that they were acquired via host-to-parasite HGT, because in each case *S. himalayana* is placed sister to, or nested within, Vitaceae with ≥70 BP. Only *atpA* from *S. himalayana* and *R. tuan-mudae* were placed elsewhere phylogenetically, sister to *Daucus* with 94 BP. Furthermore, for six of these 14 genes (i.e., *atpB*, *atpI*, *psaB*, *psbA*, *psbC*, and *psbD*), the transgenic sequences from *S. himalayana* were sister to the mitochondrial and not the plastid homologs from Vitaceae (Figure S1D). These six genes, plus three additional plastid genes (i.e., *atpA*, *ndhB*, and *rbcL*), have been shown to be incorporated into the mitochondrial genome of *Vitis* [23]. Together, these results suggest that the majority of these plastid genes were likely acquired via HGT from the host mitochondrial genome, instead of from its plastid genome.

Finally, seven of our assembled contigs demonstrated that synteny was maintained between transgenes from Rafflesiaceae and genes from the close relative of their hosts, *Vitis*, whose mitochondrial and plastid genomes were both fully annotated (i.e., *rps7*+*nad5* exon A, *psbC*+*psbD*, *sdh3*+*rpl5*+*rps14*+*cob*+*cox1*, *rpoC1*+*rpoC2*, and *sdh4+cox3*; Figure 2 and Figure S3). This, combined with our finding that two transgenes bear introns (i.e., *cox1* and *rpl2*), firmly supports our previous suggestion that transgenes in Rafflesiaceae are likely transferred as larger DNA fragments versus shorter mRNAs [28].

### Transgenes are expressed in Rafflesiaceae

Most previously reported mitochondrial transgenes in plants appear to be non-functional, i.e., they have been shown to be either introns (e.g., [3], [6], [8], [46], [47]) or pseudogenes (e.g., [7], [9]). However, among all mitochondrial transgenes identified here, six of seven sequences in *R. cantleyi* and *R. tuan-mudae*, and 13 of 16 sequences in *S. himalayana* maintain their reading frames (Table 1). To further understand if these transgenes are expressed, we re-examined the recently published transcriptome of *R. cantleyi* [28] to quantify gene expression levels of these mitochondrial genes. Our results indicate that all transgenes in *R. cantleyi* show evidence of expression (Table 1). Furthermore, although native genes in *R. cantleyi* show higher overall levels of gene expression than transgenes (Figure S4), this difference is not significant (p-value = 0.19, Welch's *t* test). Thus, transgenes are actively transcribed in this species, suggesting that they have functional promoters and likely play a role in cellular function.

### Timing of HGT events

Our broad phylogenomic assessment of mitochondrial genome provides a unique opportunity to determine if HGT we identified in Rafflesiaceae is relatively ancient or more recent. For five genes that show evidence of HGT (i.e., *atp1*, *atp4*, *cox1*, *rps7*, and *atpA*; Figure S1A and S1D), it is most parsimonious to infer that they each result from an ancient HGT event. The more ancient origin is supported by the fact that transgenic sequences from *Rafflesia* and *Sapria* form a clade. Furthermore, we found that some of these transgenes maintained synteny between *Rafflesia* and *Sapria* (e.g., *atp1* and *rps7*; Figure 2A and 2B). Therefore, these gene transfers appear to have been relatively ancient and likely occurred after the origin of stem group Rafflesiaceae (95% highest posterior density [HPD] interval of 83.1–109.5 Ma; [17], [48]) and before the origin of crown group Rafflesiaceae (69.5–95.9 Ma; [17], [48]). Both of these estimated clade ages, accounting for 95% HPD intervals, are outside the age of stem group *Tetrastigma* (36.4–65.3 Ma; [49]), and well outside the age of crown group *Tetrastigma* (25.7–49.3 Ma; [49]) (Figure 1B).

This raises the distinct possibility that Rafflesiaceae has had former host associations with other plant lineages (perhaps within Vitaceae, but also outside of the family), which may have served as past donors of transgenes. We have previously referred to this as the ghost of HGT's past [50]. In support of this possibility, none of the more ancient transgenic sequences we identified grouped with their current hosts *Tetrastigma*, as would be expected if these species served as hosts. Two genes (i.e., *atp1* and *rps7*) involved in these more ancient HGT events are sister to Vitaceae, suggesting that close relatives of *Tetrastigma* may have served as past transgenic donors. In three other cases (i.e., *atp4*, *cox1*, and *atpA*), however, transgenic sequences do not group closely to Vitaceae (e.g., Cucurbitaceae and *Daucus*; Table 1), indicating different transgenic donors (no evidence of gene conversion, which would confound phylogenetic placements, was detected in these genes using the OrgConv package [51] with p-value<0.001). To our knowledge, this is the first evidence that Rafflesiaceae may have previously parasitized different host species, which served as transgenic donors in the past. Further taxon sampling of these genes by co-authors Z.X. and Y.W. is underway and should allow us to determine those previous host donors more precisely.

For the remaining 28 instances of HGT, it is most likely that these were the result of more recent gene transfers. Transgenic sequences in these cases are found exclusively in either *Rafflesia* (i.e., *rps4*) or *Sapria* (e.g., *atp9* and *cob*), or if identified in both *Rafflesia* and *Sapria* they do not form a clade (i.e., *rpl2* and *rps1*) (Figure S1A and S1D). Evidence of such recent HGT is especially prevalent in *S. himalayana*: 17 of its transgenic sequences are sister to, or nested within, *Tetrastigma* (Table 1). Moreover, our phylogenetic analyses indicate that some of these sequences may have resulted from multiple independent gene transfers involving the same gene because transgenic sequences from Rafflesiaceae do not form a clade. In some cases, these gene transfers appear to involve multiple transgenic sequences within a single species for the same gene (i.e., *cox1* in *Sapria*, which possesses two distinct transgenic sequences that appear to have been transferred independently). In other cases, gene transfers involve multiple transgenic sequences in different species for the same gene (i.e., *rpl2* and *rps1*, which show independent transfer events for *Rafflesia* and *Sapria*). These more recent HGT events are further supported by synteny: transgenic sequences involving the same gene from *Rafflesia* and *Sapria* are located at different positions in the mitochondrial genome (e.g., *rpl2*; Figure 2A and 2C). Why some genes exhibit repeated HGT is fertile ground for future investigation.

### Conclusion

Our study is the first to comprehensively assess the magnitude of HGT in plants involving a genome (i.e., mitochondria) and a species interaction (i.e., parasitism) where it has been hypothesized to be potentially rampant. These results reveal a high degree of HGT in the mitochondrial genome of Rafflesiaceae involving genes that were likely acquired from its host at various time intervals. We previously established that in *R. cantleyi*, about 2.1% of nuclear gene transcripts have likely been acquired from its host via HGT [28]. In contrast, our study conservatively indicates that 24–41% of the mitochondrial gene sequences show evidence of HGT in Rafflesiaceae, depending on the species. These results establish for the first time that although the magnitude of HGT involving nuclear genes is appreciable, HGT involving mitochondrial genes in these parasitic plants is an order of magnitude higher. This elevated rate of HGT involving the mitochondrial genome may represent a more general pattern for other parasitic plant clades, and perhaps more broadly for angiosperms.

## Materials and Methods

### Molecular techniques and next-generation sequencing

For *R. cantleyi* and *S. himalayana*, mitochondria were isolated from ∼30 grams of fresh material from flower buds using the sucrose gradient centrifugation protocols of Jansen et al. [30]. DNA extracted from purified mitochondria was amplified with the REPLI-g Midi Kit (Qiagen, Inc.). When we were unable to acquire fresh material, total genomic DNA (gDNA) was extracted from silica-dried material using the DNeasy Plant Mini kit (Qiagen, Inc.), and treated with RNase A at 60°C for 1.5 hours to remove any residual RNA contamination. For each species, an Illumina library with the insert size of 350±50 bp was prepared from five micrograms of DNA following the protocols of Bentley et al. [52]. All libraries were sequenced on the Genome Analyzer II (Illumina, Inc.) with 100 bp paired-end runs at the FAS Center for Systems Biology at Harvard University (Table S1).

### Sequence assembly and alignment

Illumina reads were assembled *de novo* in ABySS v1.2.1 [53] using default parameters (Table S1). The assembled contigs were annotated against published mitochondrial and plastid genomes from 27 seed plants (Table S3) with BLASTN v2.2.23 [54] using an *e*-value ≤10^−5^. Gene sequences from all species were then queried against themselves using BLASTN v2.2.23. BLASTN hits with an *e*-value ≤10^−10^ were passed to MCL v08-312 [55] for Markov clustering. Only those gene clusters that included at least *Cycas*/*Spirodela* (for outgroup rooting), *Rafflesia*/*Sapria* (sequences under investigation), *Ricinus* (close relative of Rafflesiaceae), and *Vitis* (close relative of *Tetrastigma*) were retained. The nucleotide sequences of each gene were first aligned using MAFFT v6.624 [56], and then manually inspected and realigned if necessary.

### Gene copy number estimation

To assess gene copy number and corresponding genomic compartment localization of our assembled gene sequences, we mapped the Illumina gDNA reads from *R. cantleyi* [28], *R. tuan-mudae*, and *S. himalayana* to gene sequences identified here and to the 1305 nuclear genes identified from *R. cantleyi* [28] and *R. tuan-mudae* [18] using Bowtie v0.12.7 [57] (Table S2). To avoid complications with intron regions, we first divided each Illumina read into multiple 25 bp fragments following Kim and Salzberg [58], and then mapped each 25-mer with zero mismatches and unique mapping.

### Phylogenetic analyses and alternative topology tests

Our ML analyses were conducted for all genes using RAxML v7.2.8 [59] with the GTR+Γ nucleotide substitution model. The best-scoring ML tree and BP for each gene were obtained using the rapid bootstrap algorithm [60] with 500 replicates (Figures S1 and S2). For *nad5*, we also performed ML analyses on three gene regions separately (i.e., *nad5* exon A, intron A/B, and exon B; Figure 2A, see also Figure S1C), which allowed us to determine the location of homologous recombination more accurately (see above).

Alternative topology tests were performed in an ML framework using the approximately unbiased (AU) test [61] as implemented in scaleboot v0.3-3 [62] (Table 1). To generate constrained ML trees for genes that show evidence of HGT, we enforced all transgenic and native (when present) sequences from Rafflesiaceae to be monophyletic with *Ricinus*, and then conducted ML searches using these constraints.

### Gene expression level analyses

To estimate the gene expression level in *R. cantleyi*, the Illumina cDNA reads from Xi et al. [28] were mapped onto the assembled *R. cantleyi* mitochondrial gene sequences using Bowtie v0.12.7 [57] as described above. cDNA reads that mapped onto each gene sequence were then summed and further normalized to reads per kilobase per million reads (RPKM [63]; Table 1, see also Figure S4).

### Contamination and the determination of HGT in Rafflesiaceae

Tremendous care was taken to avoid and/or detect host or lab contamination during our sample preparation and data analyses. First, our DNA sample preparation and genome library sequencing of Rafflesiaceae were performed separate from any work involving *Tetrastigma*; thus, laboratory contamination of our Rafflesiaceae DNAs with *Tetrastigma* is unlikely. Second, the plastid genome has apparently been lost in Rafflesiaceae [31]. If there were any host contamination, the host's plastid gene sequences should be easily detected in our sequence data. This was not the case. Third, the mitochondrial genome sequences of *R. cantleyi* and *R. tuan-mudae* were generated from two different sources, i.e., a fresh flower bud using sucrose gradient centrifugation and silica-dried perigone lobes using total gDNA extraction, respectively (Table S1). If one of these samples, or genome libraries, were contaminated, we would not expect to have identified the identical set of transgenes from these samples. Similarly, for *S. himalayana*, all transgenes identified from the genome library prepared using sucrose gradient centrifugation were verified in our second library of this species that was prepared from total gDNA (Table S2). Fourth, most transgenic sequences identified here possess some amount of sequence divergence when directly compared with homologs from their current host. For example, all 15 transgenic sequences from *Rafflesia* show some degree of sequence divergence when directly compared with homologs from their host species, *T. rafflesiae* (mean DNA sequence distance = 0.042189). Similarly, 26 of 30 transgenic sequences from *Sapria* show some degree of sequence divergence when directly compared with homologs from their host species, *T. cruciatum* (mean DNA sequence distance = 0.020265) (Figure S1A and S1D). These sequence distances are significantly greater (p-value<0.01, Welch's *t* test) than those between the two included *Tetrastigma* species (mean DNA sequence distance = 0.001984). This is despite the fact that these two *Tetrastigma* species have diverged from each other at least 10 Ma [49]. Furthermore, three transgenic sequences from *Rafflesia* and 13 transgenic sequences from *Sapria* contain nonsense mutations (Table 1). These results strongly indicate that some period of evolution has elapsed since the time of HGT. Fifth, all seven transgenes from *R. cantleyi* show evidence of gene expression based on its transcriptome (Table 1), and levels of expression are not significantly different between transgenes and native genes (p-value = 0.19, Welch's *t* test; Figure S4). Lastly, and perhaps most importantly, seven of our assembled contigs contain both transgenes and native genes (Figure 2) indicating that these transgenes are clearly integrated into the mitochondrial genome of Rafflesiaceae.

## Supporting Information

Figure S1 Phylograms of all horizontally transferred genes in the mitochondrial genomes of *Rafflesia cantleyi*, *Rafflesia tuan-mudae*, and *Sapria himalayana*. Maximum likelihood bootstrap percentages (BP) were summarized from 500 bootstrap replicates, and only BP values greater than 50% are shown. Gene sequences from Rafflesiaceae and the host *Tetrastigma* are highlighted in red and blue, respectively. H and V indicate sequences of horizontal and vertical transmission, respectively. Number of aligned characters (chars) and scale bar (substitutions per site) are shown for each gene. (A) Phylograms for the 16 mitochondrial genes where HGT was detected. (B) Phylograms of the four mitochondrial genes with RNA editing sites excluded from our alignments. (C) Phylograms for the three gene regions of *nad5*: exons A and B and intron A/B. (D) Phylograms for the 14 genes of plastid origin where HGT was detected.
(PDF)

Figure S2 Phylograms of all vertically transferred and unassigned genes in the mitochondrial genomes of *Rafflesia cantleyi*, *Rafflesia tuan-mudae*, and *Sapria himalayana*. Maximum likelihood bootstrap percentages (BP) were summarized from 500 bootstrap replicates, and only BP values greater than 50% are shown. Gene sequences from Rafflesiaceae and the host *Tetrastigma* are highlighted in red and blue, respectively. V indicates sequences of vertical transmission. Number of aligned characters (chars) and scale bar (substitutions per site) are shown for each gene.
(PDF)

Figure S3 Gene organization of three assembled contigs (A–C) for *Sapria himalayana* (Rafflesiaceae) and *Vitis vinifera* (Vitaceae). The red boxes indicate *Vitis*-like genes. Pseudogenes are represented by striped boxes, and the sequence length (in kilobases [kb]) is indicated to the right of each assembled contig.
(PDF)

Figure S4 Boxplot of gene expression levels of horizontally (HGT) and vertically (VGT) transferred mitochondrial gene sequences in *Rafflesia cantleyi*. The number of gene sequences for each category is shown in parentheses; RPKM = reads per kilobase per million reads.
(PDF)

Table S1 Newly sequenced species in this study with associated assembly statistics.
(PDF)

Table S2 Estimated gene copy number for *Rafflesia cantleyi*, *Rafflesia tuan-mudae*, and *Sapria himalayana*. The Illumina genomic DNA reads from *R. cantleyi*, *R. tuan-mudae*, and *S. himalayana* were mapped onto the 38 mitochondrial genes, 14 genes of plastid origin, and 1,305 nuclear genes from *R. cantleyi* and *R. tuan-mudae*. Illumina reads that mapped onto each gene were then summed and further normalized to reads per base pair.
(XLS)

Table S3 Data sources of gene sequences included in our phylogenetic analyses.
(PDF)
