# Supplementary material for: Massive Mitochondrial Gene Transfer in a Parasitic Flowering Plant Clade
Source: PLoS Genet. 2013 Feb 14;9(2):e1003265. doi: 10.1371/journal.pgen.1003265 (PMC3573108; doi:10.1371/journal.pgen.1003265)
Supplement: Figure S1 — Phylograms of all horizontally transferred genes in the mitochondrial genomes of Rafflesia cantleyi, Rafflesia tuan-mudae, and Sapria himalayana. Maximum likelihood bootstrap percentages (BP) were summarized from 500 bootstrap replicates, and only BP values greater than 50% are shown. Gene sequences from Rafflesiaceae and the host Tetrastigma are highlighted in red and blue, respectively. H and V indicate sequences of horizontal and vertical transmission, respectively. Number of aligned characters (chars) and scale bar (substitutions per site) are shown for each gene. (A) Phylograms for the 16 mitochondrial genes where HGT was detected. (B) Phylograms of the four mitochondrial genes with RNA editing sites excluded from our alignments. (C) Phylograms for the three gene regions of nad5: exons A and B and intron A/B. (D) Phylograms for the 14 genes of plastid origin where HGT was detected. (PDF) [file pgen.1003265.s001.pdf]

Figure S1. Phylograms of all horizontally transferred genes in the mitochondrial genomes of *Rafflesia cantleyi*, *Rafflesia tuan-mudae*, and *Sapria himalayana*. Maximum likelihood bootstrap percentages (BP) were summarized from 500 bootstrap replicates, and only BP values greater than 50% are shown. Gene sequences from Rafflesiaceae and the host *Tetrastigma* are highlighted in red and blue, respectively. H and V indicate sequences of horizontal and vertical transmission, respectively. Number of aligned characters (chars) and scale bar (substitutions per site) are shown for each gene. (A) Phylograms for the 16 mitochondrial genes where HGT was detected. (B) Phylograms of the four mitochondrial genes with RNA editing sites excluded from our alignments. (C) Phylograms for the three gene regions of *nad5*: exons A and B and intron A/B. (D) Phylograms for the 14 genes of plastid origin where HGT was detected.

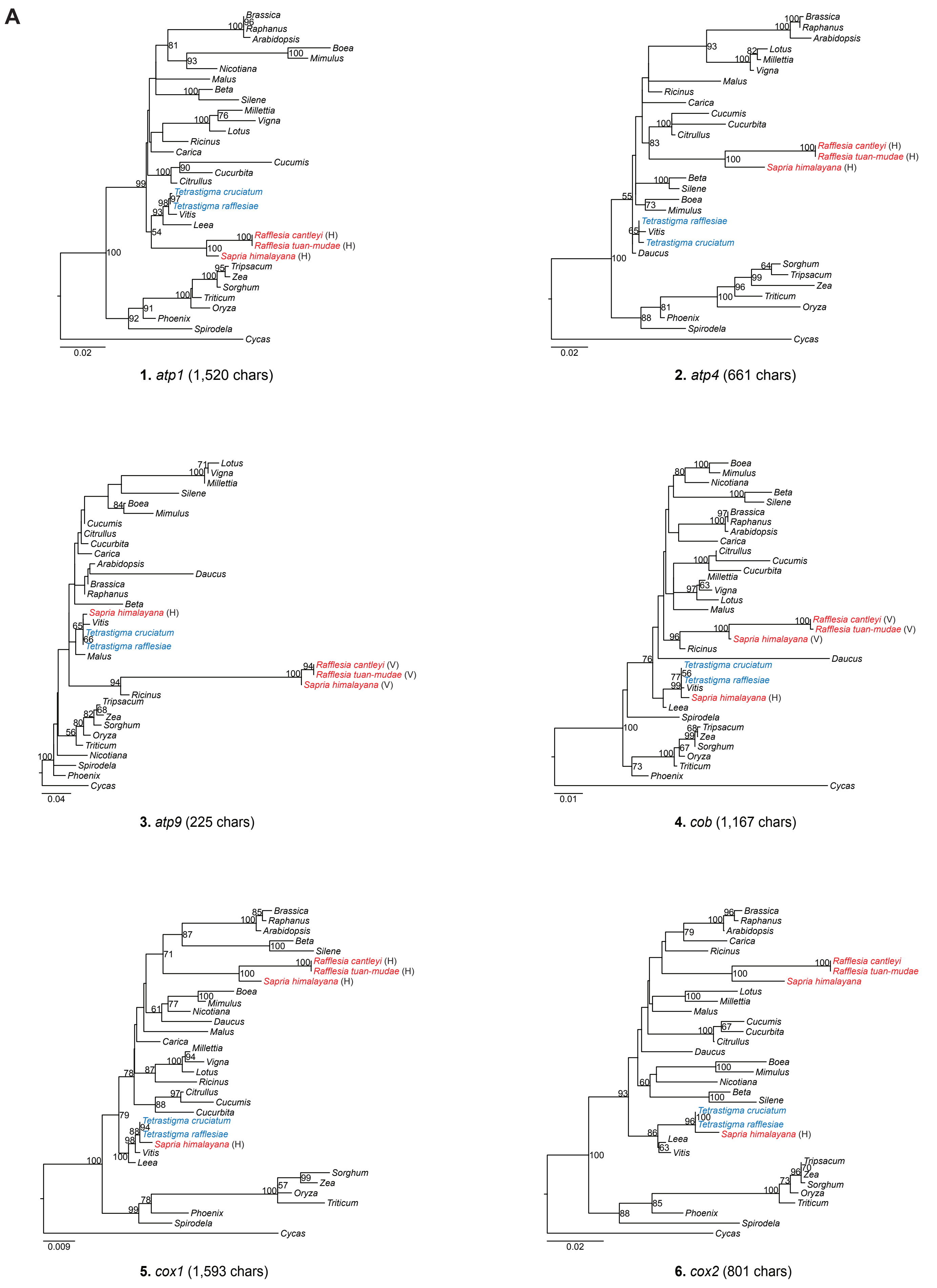
A
Brassica
Raphanus
Arabidopsis
Boea
Mimulus
Nicotiana
Malus
Beta
Silene
Millettia
Vigna
Lotus
Ricinus
Carica
Cucumis
Cucurbita
Citrullus
Tetrastigma cruciatum
Tetrastigma rafflesiae
Vitis
Leea
Rafflesia cantleyi (H)
Rafflesia tuan-mudae (H)
Sapria himalayana (H)
Tripsacum
Zea
Sorghum
Triticum
Oryza
Phoenix
Spirodela
Cycas
0.02
1. atp1 (1,520 chars)
Brassica
Raphanus
Arabidopsis
Lotus
Millettia
Vigna
Malus
Ricinus
Carica
Cucumis
Cucurbita
Citrullus
Rafflesia cantleyi (H)
Rafflesia tuan-mudae (H)
Sapria himalayana (H)
Beta
Silene
Boea
Mimulus
Tetrastigma rafflesiae
Vitis
Tetrastigma cruciatum
Daucus
Sorghum
Tripsacum
Zea
Triticum
Oryza
Phoenix
Spirodela
Cycas
0.02
2. atp4 (661 chars)
Lotus
Vigna
Millettia
Silene
Boea
Mimulus
Cucumis
Citrullus
Cucurbita
Carica
Arabidopsis
Daucus
Brassica
Raphanus
Beta
Sapria himalayana (H)
Vitis
Tetrastigma cruciatum
Tetrastigma rafflesiae
Malus
Rafflesia cantleyi (V)
Rafflesia tuan-mudae (V)
Sapria himalayana (V)
Ricinus
Tripsacum
Zea
Sorghum
Oryza
Triticum
Nicotiana
Spirodela
Phoenix
Cycas
0.04
3. atp9 (225 chars)
Boea
Mimulus
Nicotiana
Beta
Silene
Brassica
Raphanus
Arabidopsis
Carica
Citrullus
Cucumis
Cucurbita
Millettia
Vigna
Lotus
Malus
Rafflesia cantleyi (V)
Rafflesia tuan-mudae (V)
Sapria himalayana (V)
Ricinus
Daucus
Tetrastigma cruciatum
Tetrastigma rafflesiae
Vitis
Sapria himalayana (H)
Leea
Spirodela
Tripsacum
Zea
Sorghum
Oryza
Triticum
Phoenix
Cycas
0.01
4. cob (1,167 chars)
Brassica
Raphanus
Arabidopsis
Beta
Silene
Rafflesia cantleyi (H)
Rafflesia tuan-mudae (H)
Sapria himalayana (H)
Boea
Mimulus
Nicotiana
Daucus
Malus
Carica
Millettia
Vigna
Lotus
Ricinus
Citrullus
Cucumis
Cucurbita
Tetrastigma cruciatum
Tetrastigma rafflesiae
Sapria himalayana (H)
Vitis
Leea
Sorghum
Zea
Oryza
Triticum
Phoenix
Spirodela
Cycas
0.009
5. cox1 (1,593 chars)
Brassica
Raphanus
Arabidopsis
Carica
Ricinus
Rafflesia cantleyi
Rafflesia tuan-mudae
Sapria himalayana
Lotus
Millettia
Malus
Cucumis
Cucurbita
Citrullus
Daucus
Boea
Mimulus
Nicotiana
Beta
Silene
Tetrastigma cruciatum
Tetrastigma rafflesiae
Sapria himalayana (H)
Leea
Vitis
Tripsacum
Zea
Sorghum
Oryza
Triticum
Phoenix
Spirodela
Cycas
0.02
6. cox2 (801 chars)

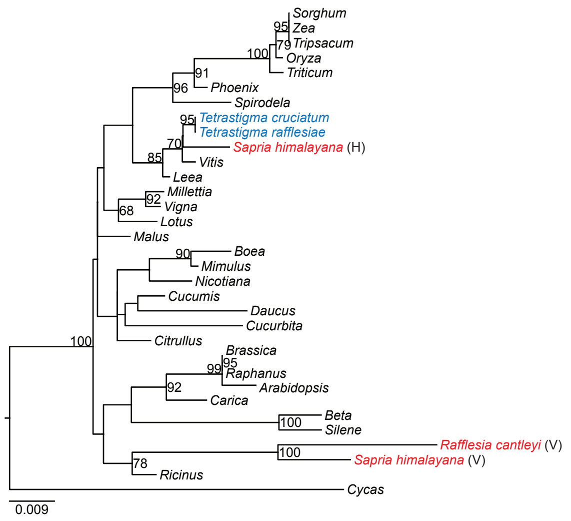

7. *cox3* (798 chars)

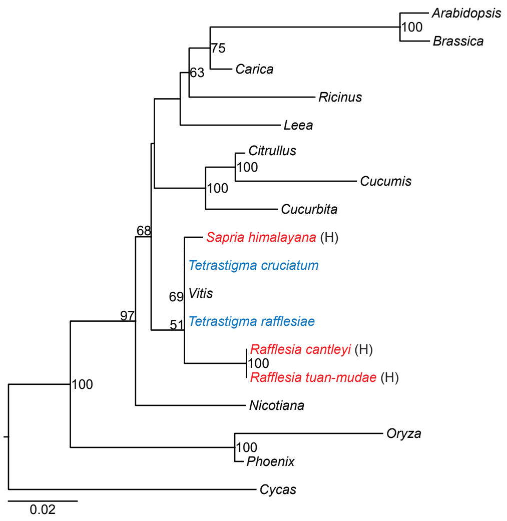

8. *rpl2* (1,244 chars)

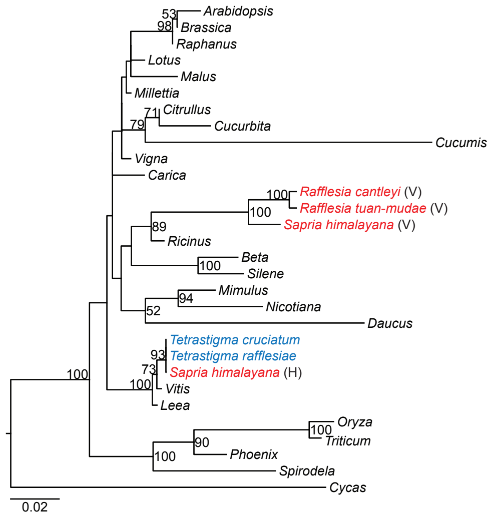

9. *rpl5* (597 chars)

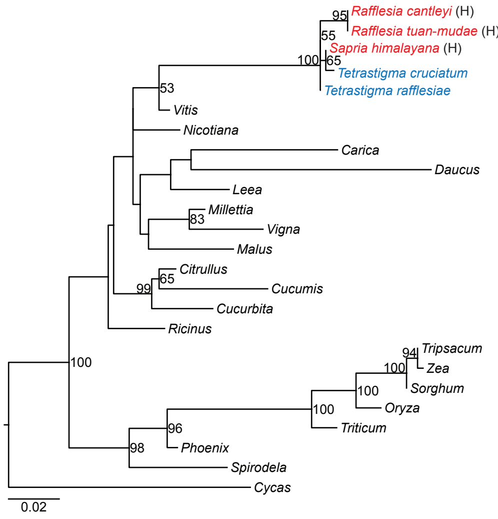

10. *rps1* (662 chars)

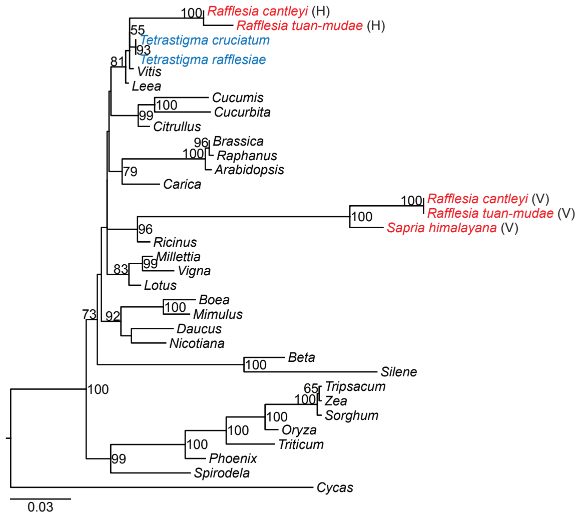

11. *rps4* (1,317 chars)

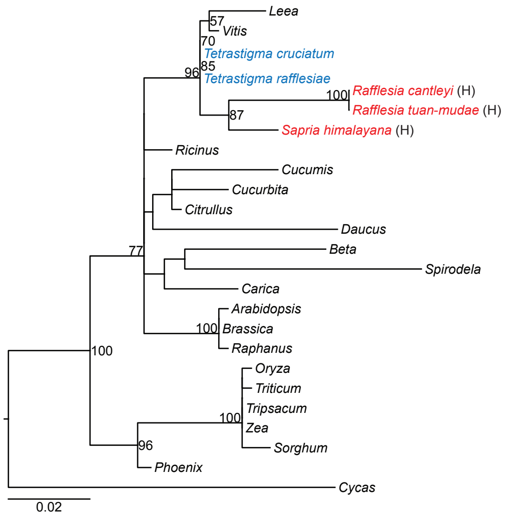

12. *rps7* (456 chars)

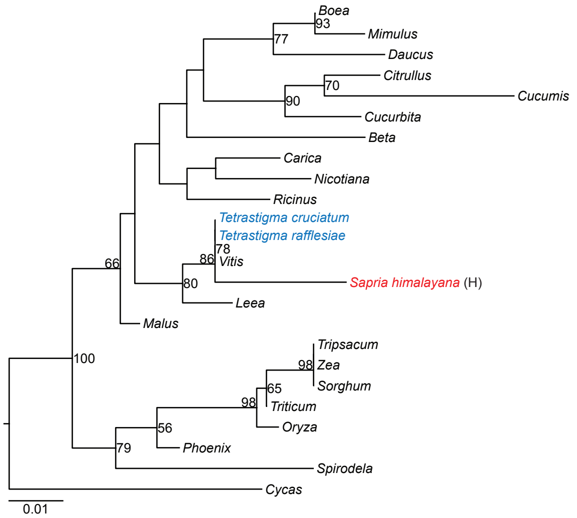

13. *rps13* (347 chars)

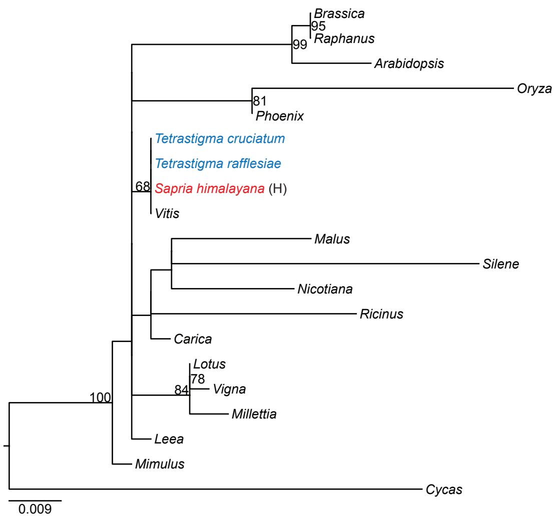

14. *rps14* (311 chars)

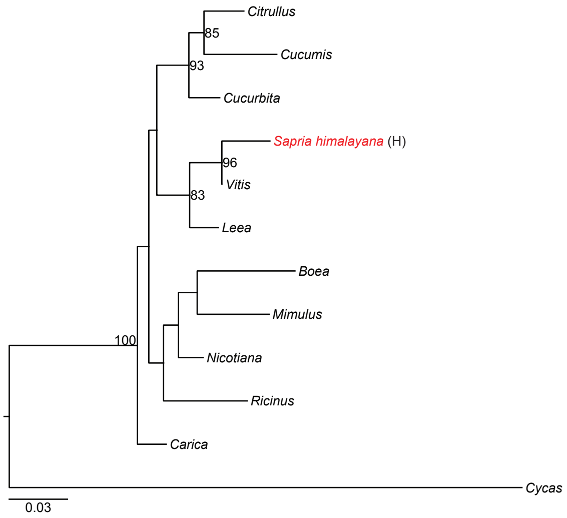

15. *sdh3* (265 chars)

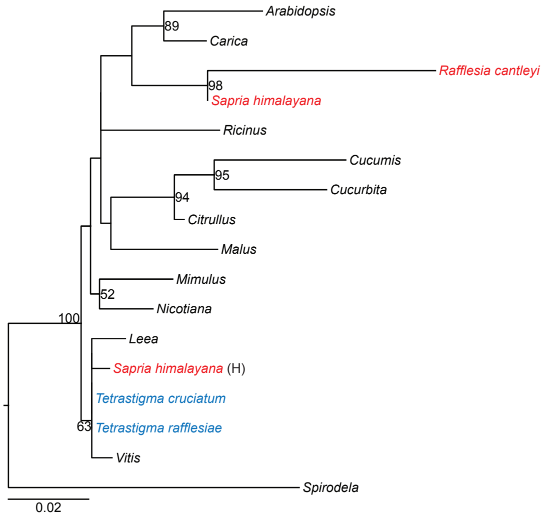

16. *sdh4* (397 chars)

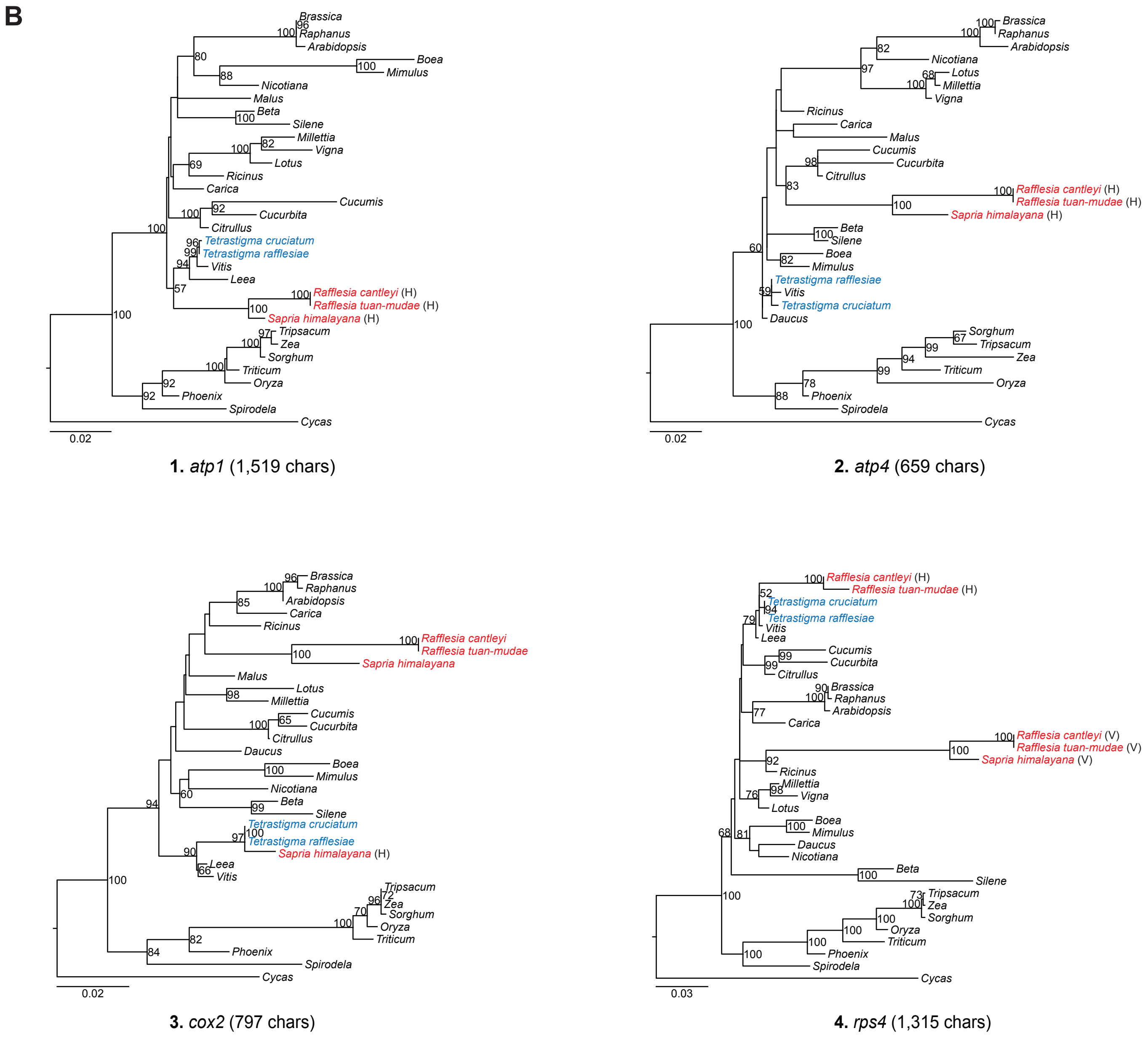
B
Brassica
Raphanus
Arabidopsis
Boea
Mimulus
Nicotiana
Malus
Beta
Silene
Millettia
Vigna
Lotus
Ricinus
Carica
Cucumis
Cucurbita
Citrullus
Tetrastigma cruciatum
Tetrastigma rafflesiae
Vitis
Leea
Rafflesia cantleyi (H)
Rafflesia tuan-mudae (H)
Sapria himalayana (H)
Tripsacum
Zea
Sorghum
Triticum
Oryza
Phoenix
Spirodela
Cycas
0.02
1. atp1 (1,519 chars)
Daucus
2. atp4 (659 chars)
Rafflesia cantleyi
Rafflesia tuan-mudae
Sapria himalayana
3. cox2 (797 chars)
Rafflesia cantleyi (V)
Rafflesia tuan-mudae (V)
Sapria himalayana (V)
0.03
4. rps4 (1,315 chars)

C

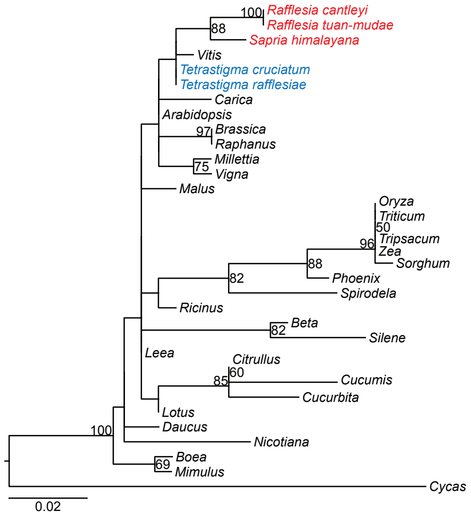

1. *nad5* exon A (230 chars)

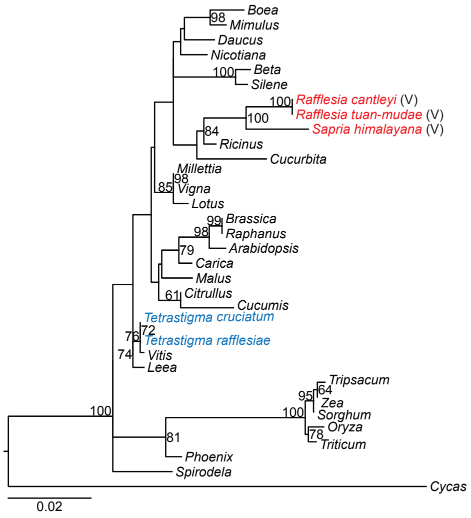

2. *nad5* exon B (1,225 chars)

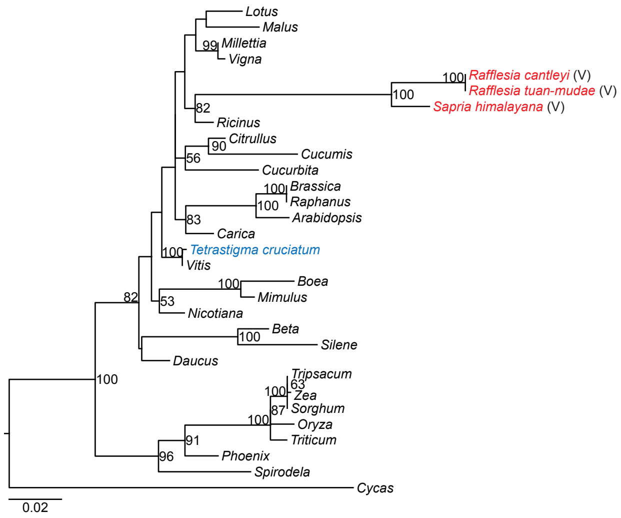

3. *nad5* intron A/B (2,014 chars)

D

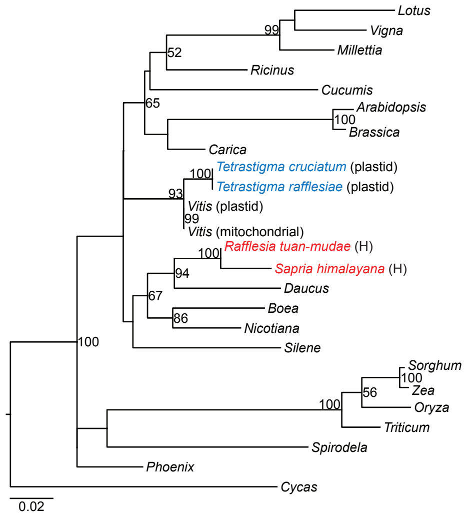

1. *atpA* (538 chars)

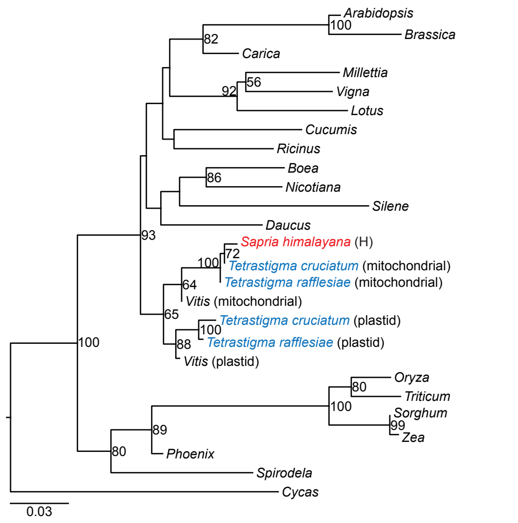

2. *atpB* (483 chars)

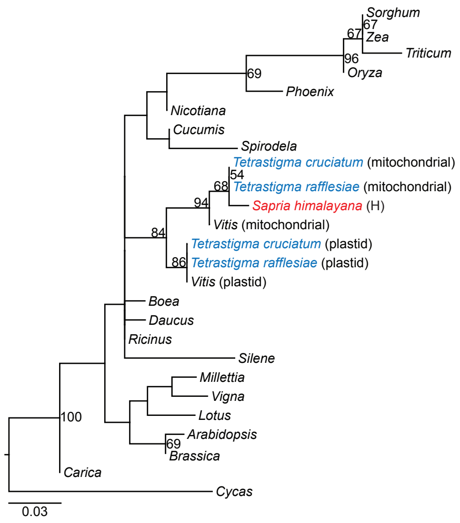

3. *atpI* (100 chars)

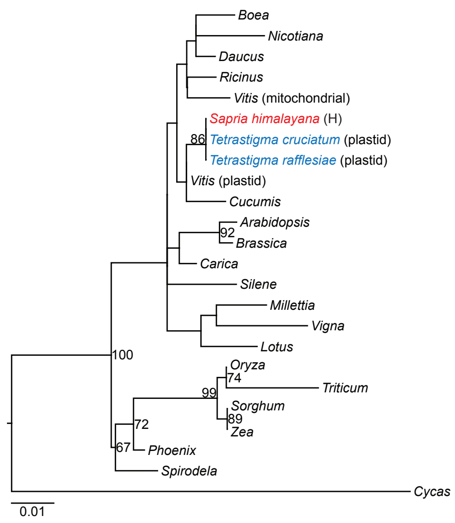

4. *ndhB* (447 chars)

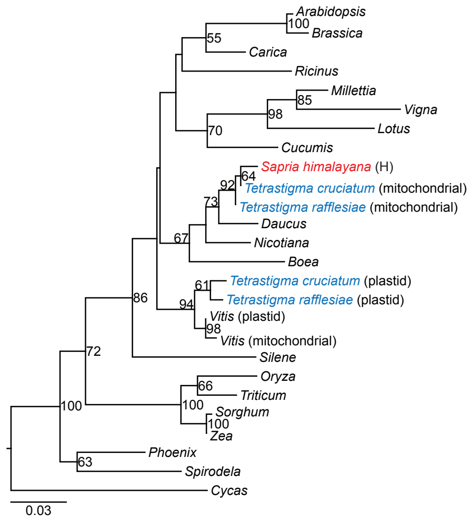

5. *psaB* (374 chars)

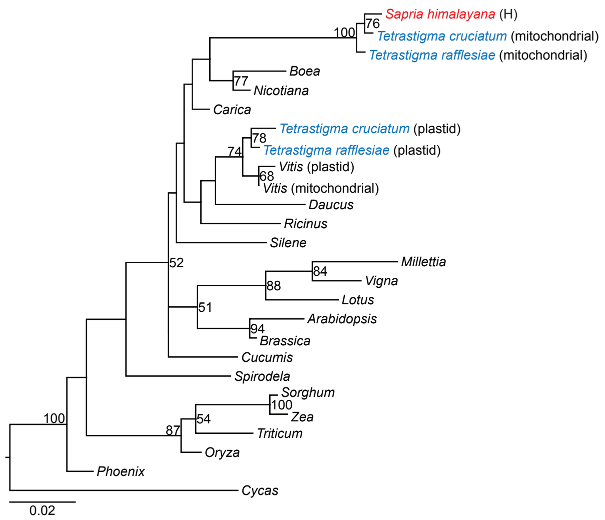

6. *psbA* (413 chars)

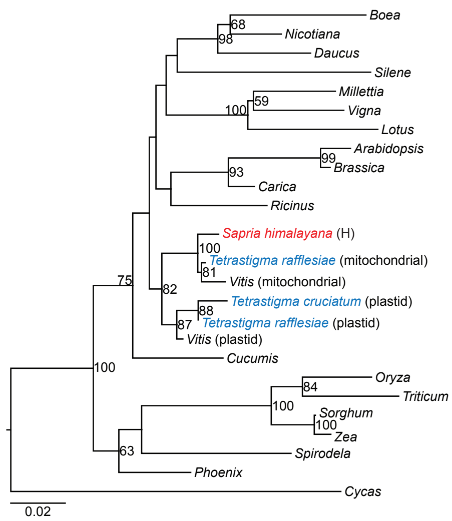

7. *psbC* (997 chars)

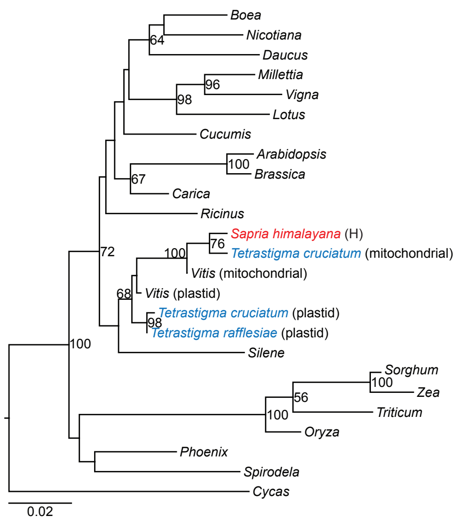

8. *psbD* (776 chars)

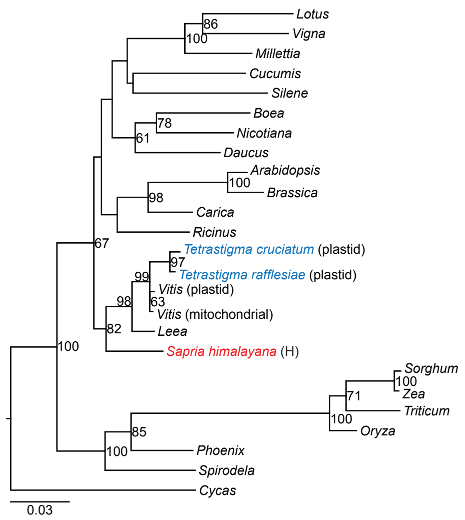

9. *rbcL* (1,396 chars)

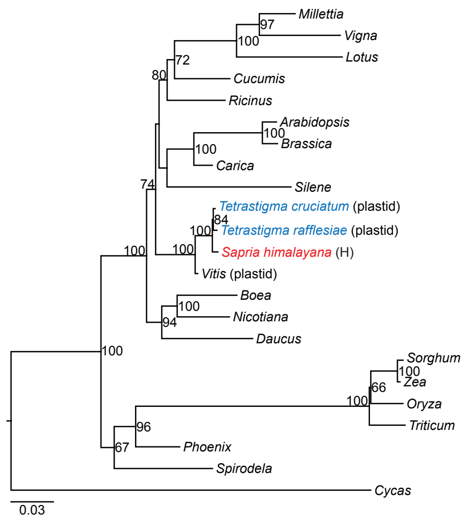

10. *rpoC1* (1,641 chars)

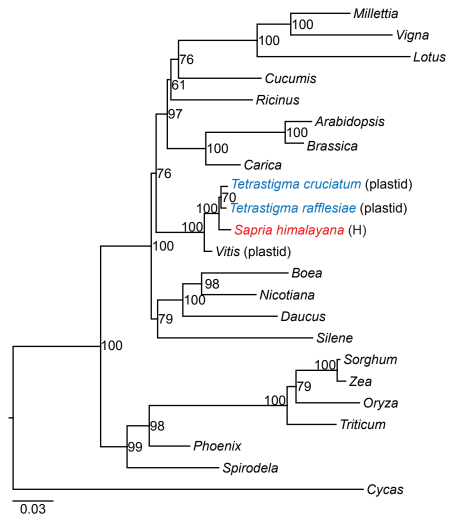

11. *rpoC2* (2,562 chars)

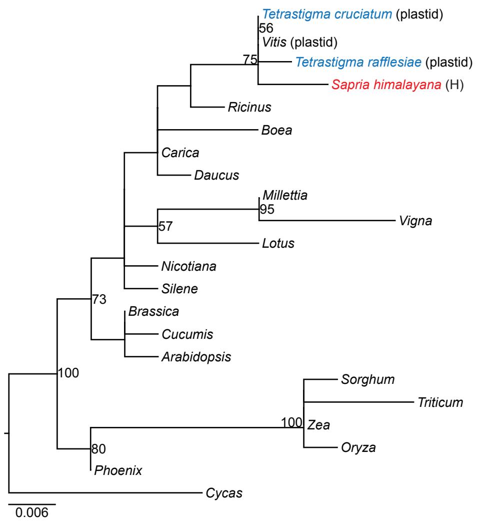

12. *rps12* (232 chars)

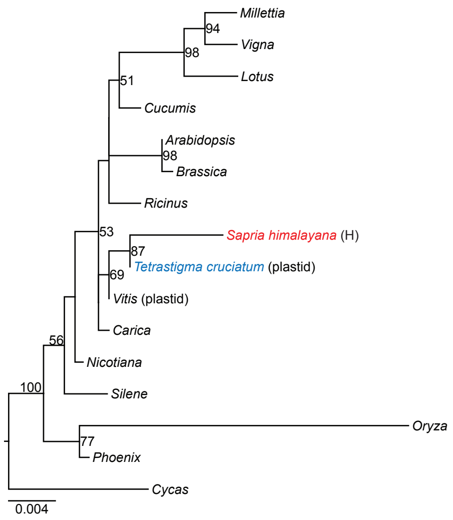

13. *rrn16* (1,146 chars)

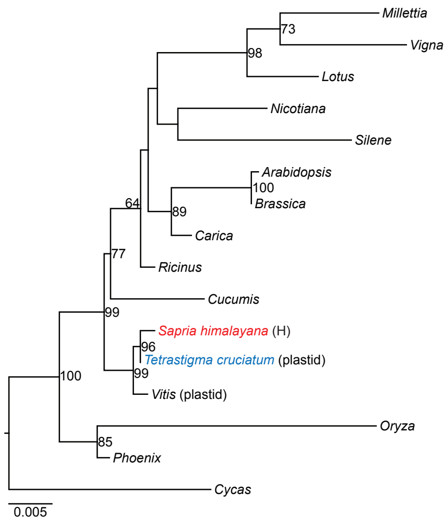

14. *rrn23* (1,441 chars)
