# Supplementary material for: Massive Mitochondrial Gene Transfer in a Parasitic Flowering Plant Clade
Source: PLoS Genet. 2013 Feb 14;9(2):e1003265. doi: 10.1371/journal.pgen.1003265 (PMC3573108; doi:10.1371/journal.pgen.1003265)
Supplement: Figure S2 — Phylograms of all vertically transferred and unassigned genes in the mitochondrial genomes of Rafflesia cantleyi, Rafflesia tuan-mudae, and Sapria himalayana. Maximum likelihood bootstrap percentages (BP) were summarized from 500 bootstrap replicates, and only BP values greater than 50% are shown. Gene sequences from Rafflesiaceae and the host Tetrastigma are highlighted in red and blue, respectively. V indicates sequences of vertical transmission. Number of aligned characters (chars) and scale bar (substitutions per site) are shown for each gene. (PDF) [file pgen.1003265.s002.pdf]

Figure S2. Phylograms of all vertically transferred and unassigned genes in the mitochondrial genomes of *Rafflesia cantleyi*, *Rafflesia tuan-mudae*, and *Sapria himalayana*. Maximum likelihood bootstrap percentages (BP) were summarized from 500 bootstrap replicates, and only BP values greater than 50% are shown. Gene sequences from Rafflesiaceae and the host *Tetrastigma* are highlighted in red and blue, respectively. V indicates sequences of vertical transmission. Number of aligned characters (chars) and scale bar (substitutions per site) are shown for each gene.

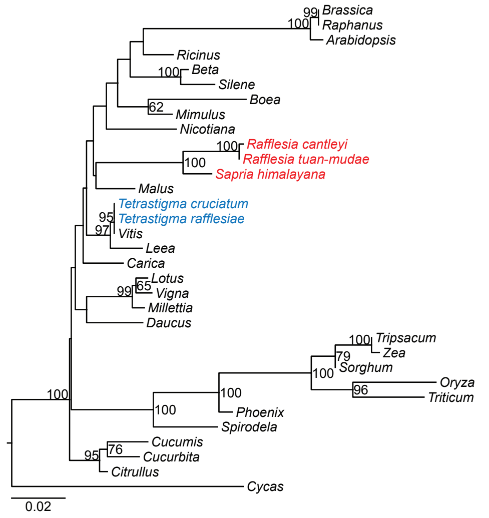

1. *atp6* (726 chars)

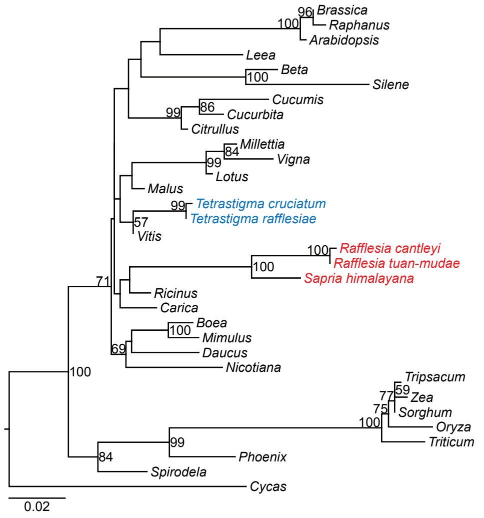

2. *atp8* (508 chars)

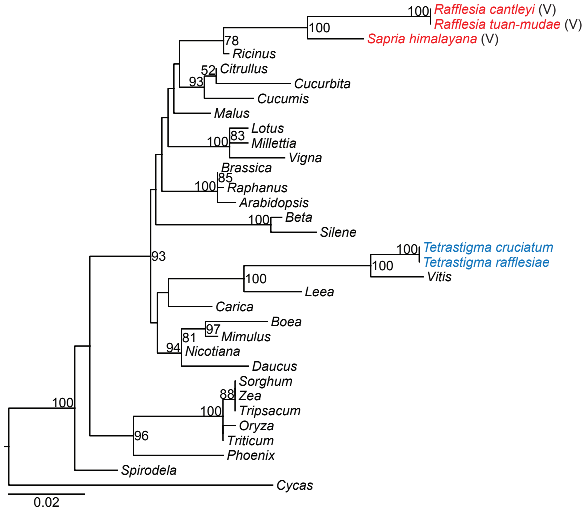

3. *ccmB* (627 chars)

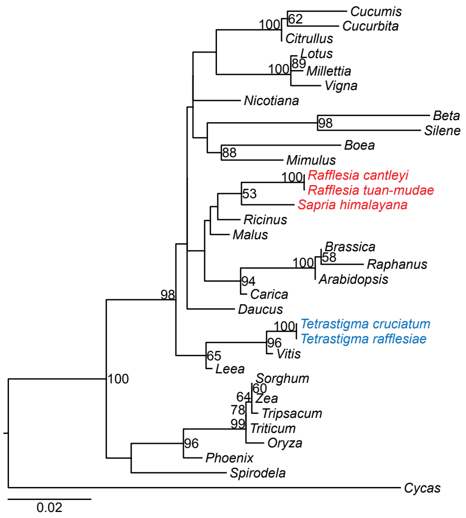

4. *ccmC* (729 chars)

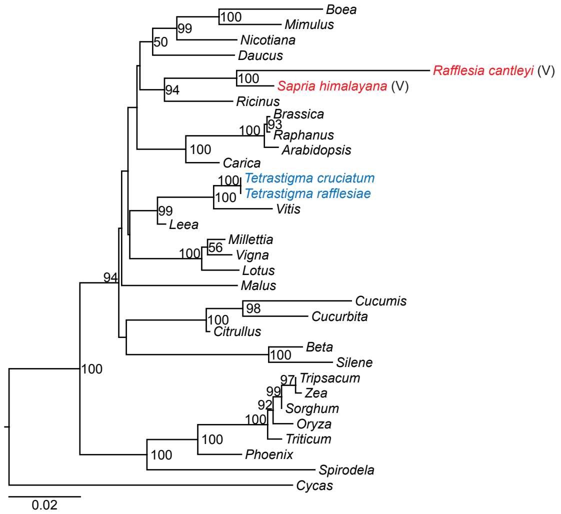

5. *ccmFC* (1,488 chars)

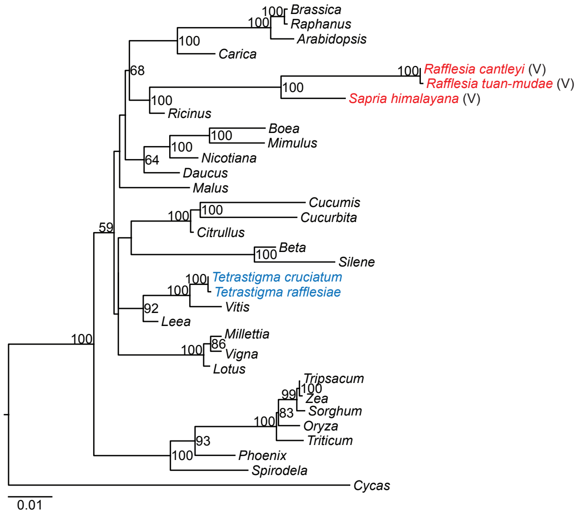

6. *ccmFN* (1,842 chars)

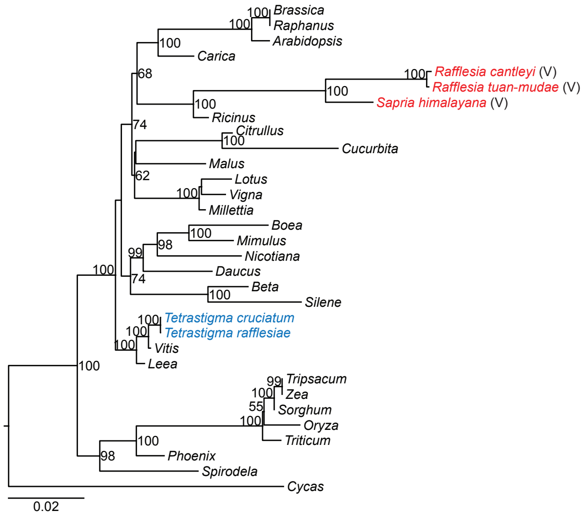

7. *matR* (2,096 chars)

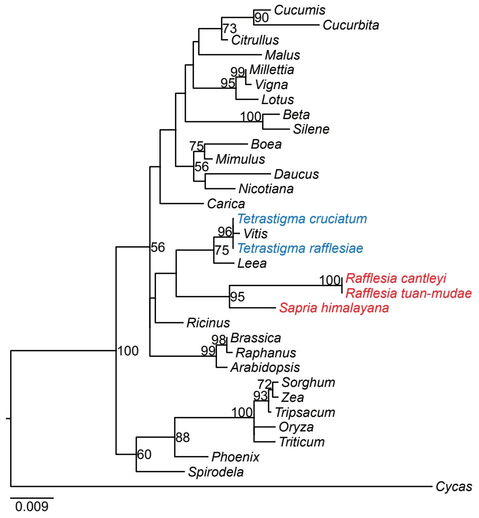

8. *nad1* (989 chars)

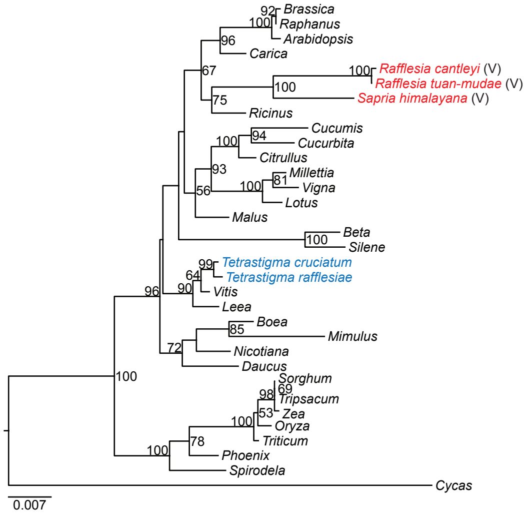

9. *nad2* (1,503 chars)

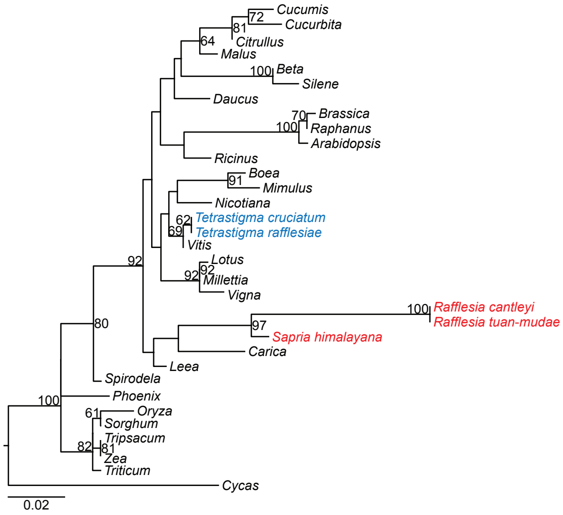

10. *nad3* (357 chars)

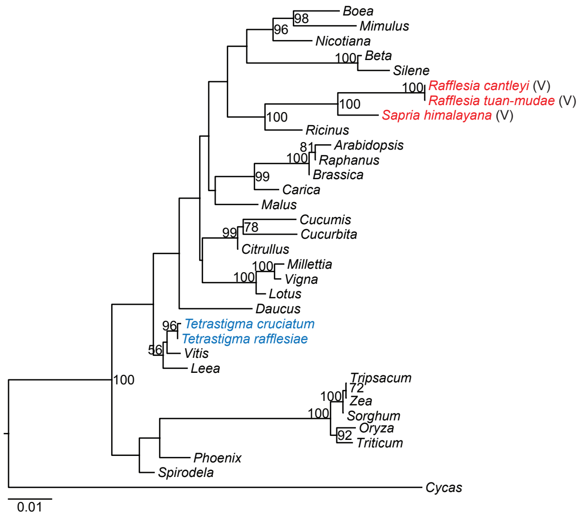

11. *nad4* (1,488 chars)

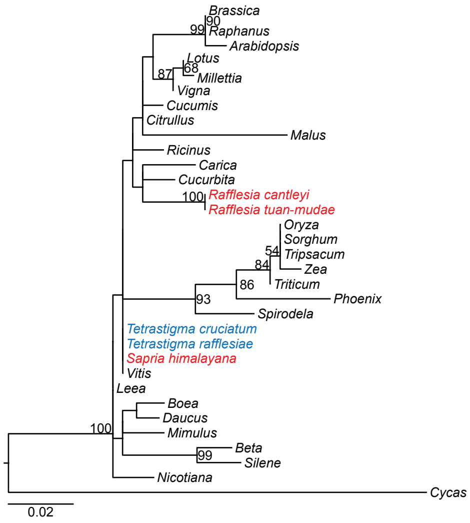

12. *nad4L* (303 chars)

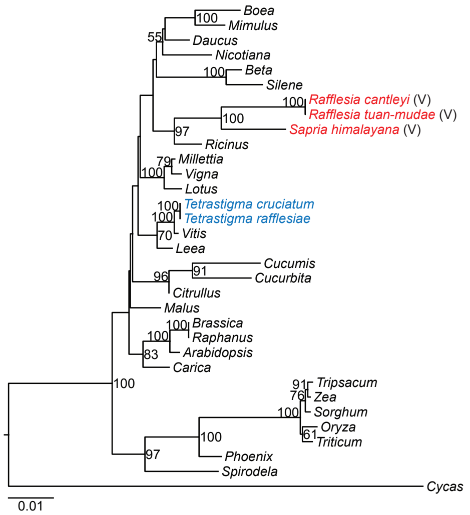

13. *nad5* (2,025 chars)

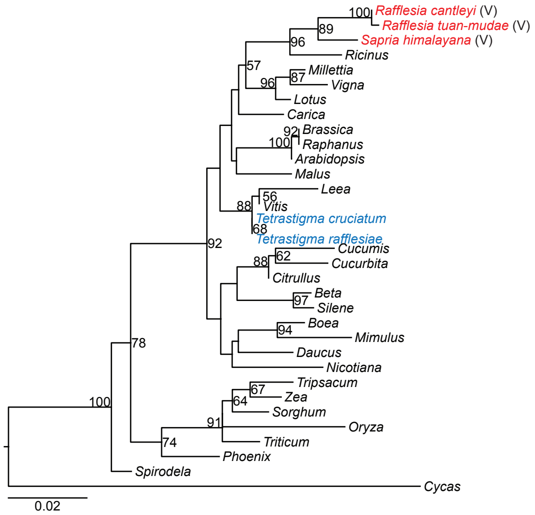

14. *nad6* (590 chars)

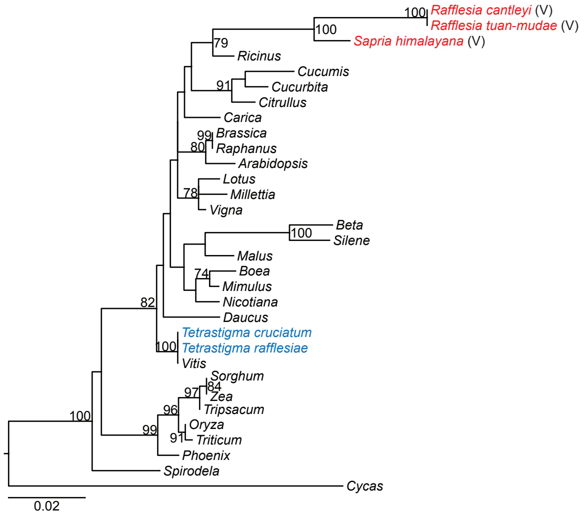

15. *nad9* (600 chars)

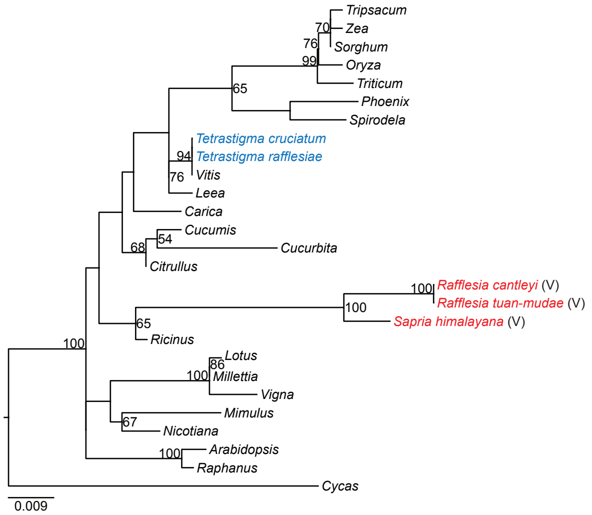

16. *rpl16* (434 chars)

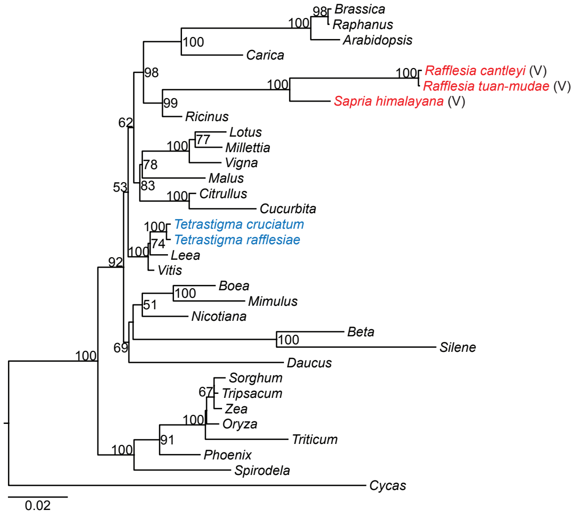

17. *rps3* (1,815 chars)

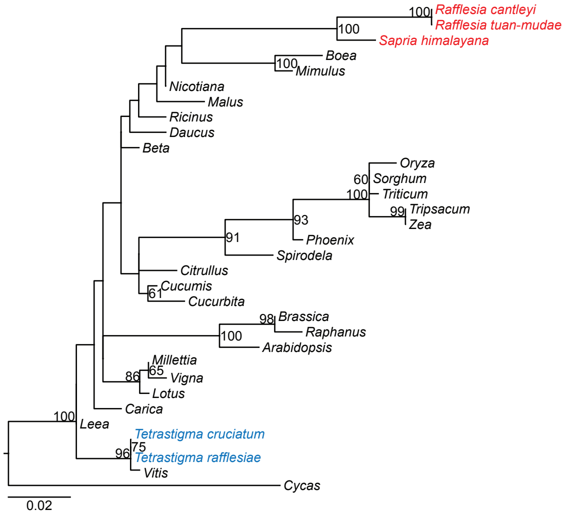

18. *rps12* (378 chars)

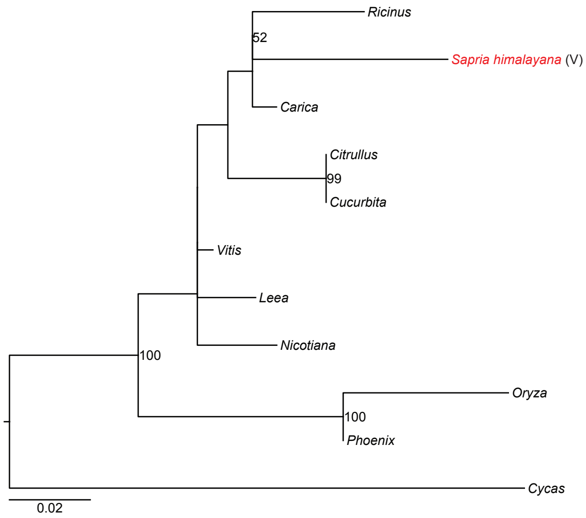

19. *rps19* (263 chars)

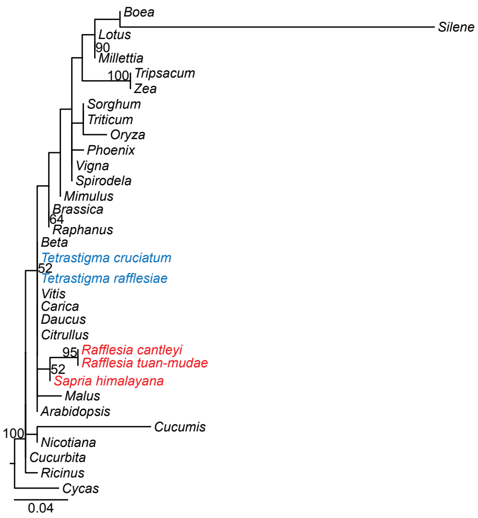

20. *rrn5* (126 chars)

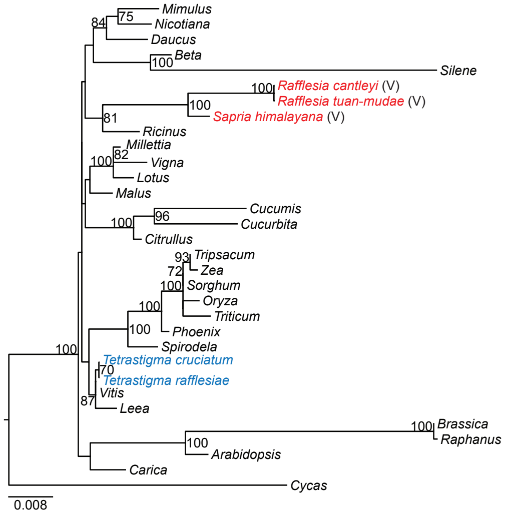

21. *rrn18* (1,813 chars)

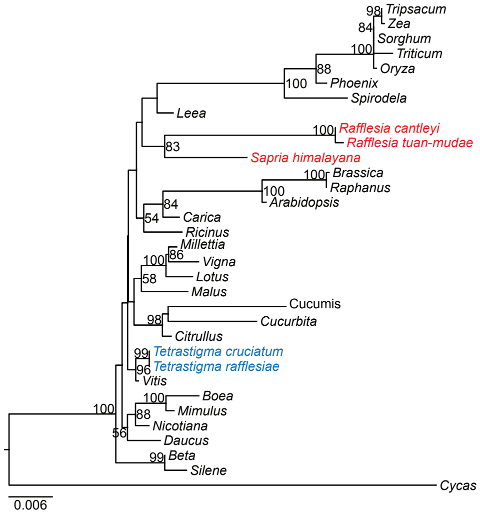

22. *rrn26* (3,017 chars)
