# Supplementary material for: Massive Mitochondrial Gene Transfer in a Parasitic Flowering Plant Clade
Source: PLoS Genet. 2013 Feb 14;9(2):e1003265. doi: 10.1371/journal.pgen.1003265 (PMC3573108; doi:10.1371/journal.pgen.1003265)
Supplement: Figure S3 — Gene organization of three assembled contigs (A–C) for Sapria himalayana (Rafflesiaceae) and Vitis vinifera (Vitaceae). The red boxes indicate Vitis-like genes. Pseudogenes are represented by striped boxes, and the sequence length (in kilobases [kb]) is indicated to the right of each assembled contig. (PDF) [file pgen.1003265.s003.pdf]

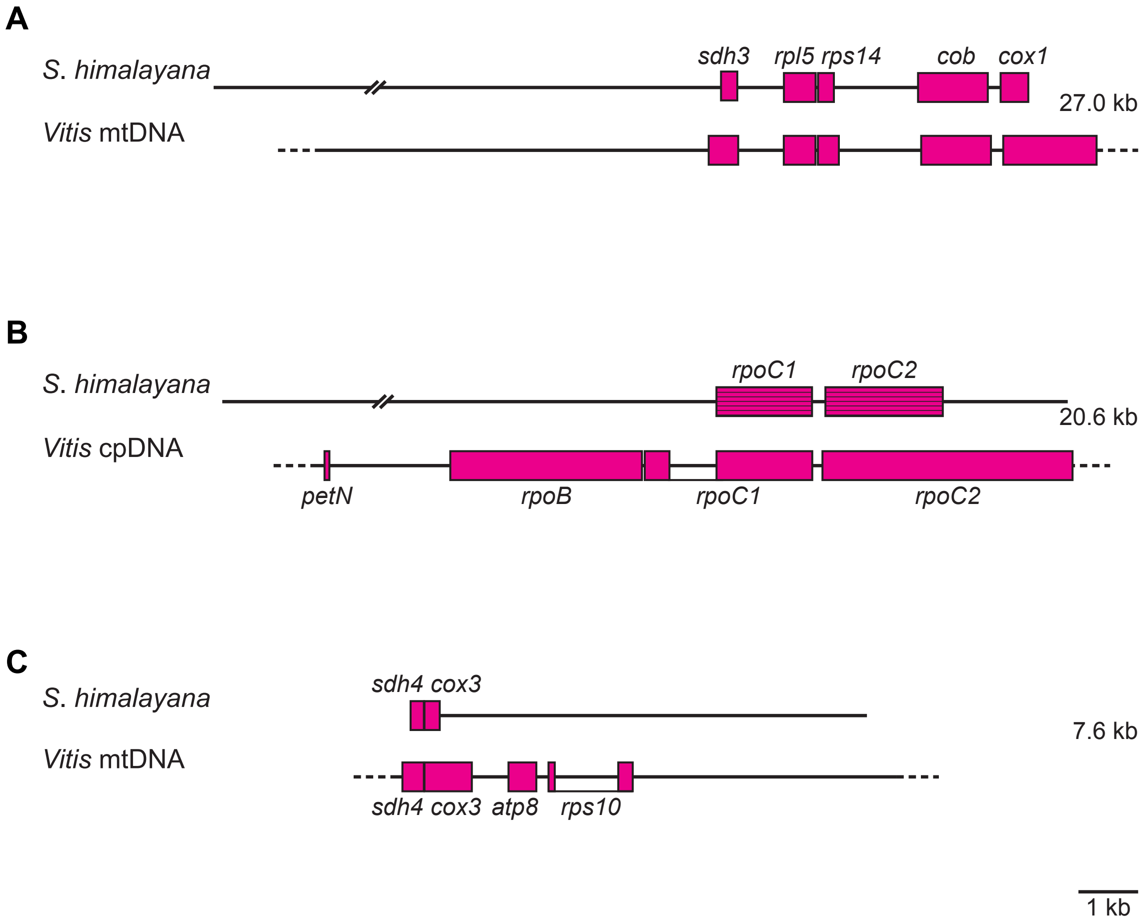

Figure S3. Gene organization of three assembled contigs (A–C) for *Sapria himalayana* (Rafflesiaceae) and *Vitis vinifera* (Vitaceae). The red boxes indicate *Vitis*-like genes. Pseudogenes are represented by striped boxes, and the sequence length (in kilobases [kb]) is indicated to the right of each assembled contig.
