# Supplementary material for: Massive Mitochondrial Gene Transfer in a Parasitic Flowering Plant Clade
Source: PLoS Genet. 2013 Feb 14;9(2):e1003265. doi: 10.1371/journal.pgen.1003265 (PMC3573108; doi:10.1371/journal.pgen.1003265)
Supplement: Figure S4 — Boxplot of gene expression levels of horizontally (HGT) and vertically (VGT) transferred mitochondrial gene sequences in Rafflesia cantleyi. The number of gene sequences for each category is shown in parentheses; RPKM = reads per kilobase per million reads. (PDF) [file pgen.1003265.s004.pdf]

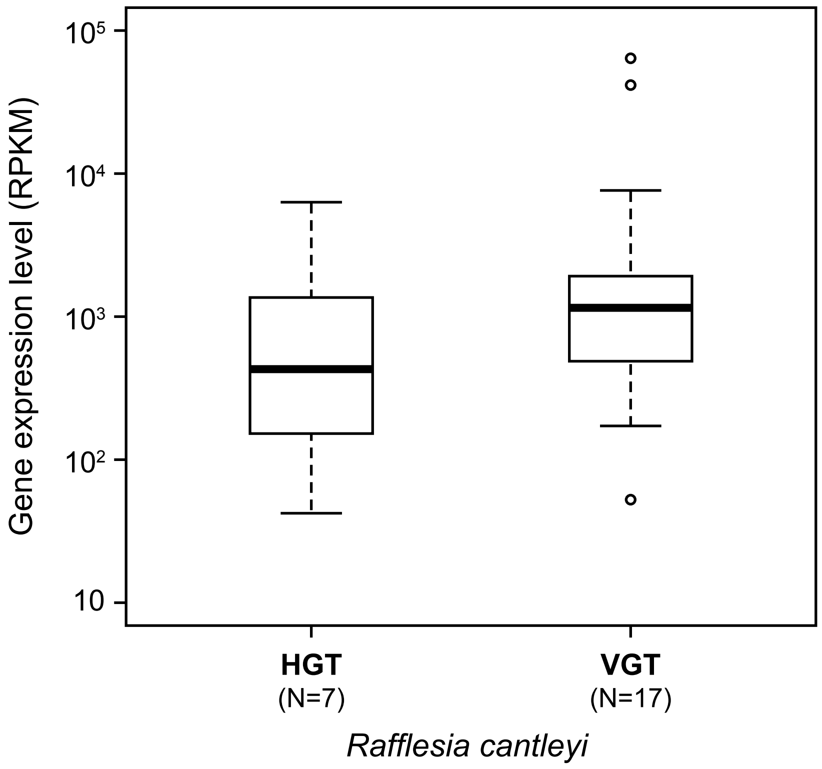

Figure S4. Boxplot of gene expression levels of horizontally (HGT) and vertically (VGT) transferred mitochondrial gene sequences in *Rafflesia cantleyi*. The number of gene sequences for each category is shown in parentheses; RPKM = reads per kilobase per million reads.
