# Supplementary material for: Massive Mitochondrial Gene Transfer in a Parasitic Flowering Plant Clade
Source: PLoS Genet. 2013 Feb 14;9(2):e1003265. doi: 10.1371/journal.pgen.1003265 (PMC3573108; doi:10.1371/journal.pgen.1003265)
Supplement: Table S1 — Newly sequenced species in this study with associated assembly statistics. (PDF) [file pgen.1003265.s005.pdf]

Table S1. Newly sequenced species in this study with associated assembly statistics

Species	Illumina library source	No. of reads per library	Max. contig length (bp)	N50 (bp)	No. of gene sequences analyzed	Average coverage for gene sequences	NCBI accession number
**Rafflesiaceae**							
Rafflesia cantleyi Solms-Laubach	Enriched mitochondria	3,325,206	18,830	9,922	35	16.1	SRR629613
Rafflesia tuan-mudae Becc.	Total genomic DNA	16,592,270	39,178	8,738	33	9.3	SRR629600
Sapria himalayana Griffith.	Enriched mitochondria	7,957,084	53,796	12,290	59	42.5	SRR629601
	Total genomic DNA	12,292,818	–	–	–	–	SRR629606
**Vitaceae**							
Leea guineensis G. Don	Total genomic DNA	7,002,750	14,273	2,516	35	2.2	SRR629604
Tetrastigma cruciatum Craib & Gagnepain	Total genomic DNA	9,845,286	81,423	3,902	50	3.6	SRR629611
Tetrastigma rafflesiae Miq.	Total genomic DNA	10,278,372	47,310	3,431	48	4.5	SRR629612
