# Supplementary material for: Massive Mitochondrial Gene Transfer in a Parasitic Flowering Plant Clade
Source: PLoS Genet. 2013 Feb 14;9(2):e1003265. doi: 10.1371/journal.pgen.1003265 (PMC3573108; doi:10.1371/journal.pgen.1003265)
Supplement: Table S3 — Data sources of gene sequences included in our phylogenetic analyses. (PDF) [file pgen.1003265.s007.pdf]

Table S3. Data sources of gene sequences included in our phylogenetic analyses.

Species	Organelle	GenBank accession number	No. of gene sequences included
Arabidopsis thaliana (L.) Heynh.	Mitochondrion	NC_001284	34
	Plastid	NC_000932	14
Beta vulgaris L.	Mitochondrion	NC_002511	31
	Plastid	–	–
Boea hygrometrica (Bunge) R. Brown	Mitochondrion	NC_016741	29
	Plastid	NC_016468	12
Brassica napus L.	Mitochondrion	NC_008285	32
	Plastid	NC_016734	14
Carica papaya L.	Mitochondrion	NC_012116	38
	Plastid	NC_010323	14
Citrullus lanatus (Thunb.) Matsum. & Nakai	Mitochondrion	NC_014043	37
	Plastid	–	–
Cucumis sativus L.	Mitochondrion	NC_016005	34
	Plastid	NC_007144	14
Cucurbita pepo L.	Mitochondrion	NC_014050	37
	Plastid	–	–
Cycas taitungensis Shen, Hill, Tsou & Chen	Mitochondrion	NC_010303	37
	Plastid	NC_009618	14
Daucus carota L.	Mitochondrion	NC_017855	31
	Plastid	NC_008325	12
Lotus japonicus L.	Mitochondrion	NC_016743	31
	Plastid	NC_002694	14
Malus domestica Borkh.	Mitochondrion	NC_018554	32
	Plastid	–	–
Millettia pinnata (L.) Panigrahi	Mitochondrion	NC_016742	32
	Plastid	NC_016708	14
Mimulus guttatus DC.	Mitochondrion	NC_018041	34
	Plastid	–	–
Nicotiana tabacum L.	Mitochondrion	NC_006581	37
	Plastid	NC_001879	14

Oryza sativa L.	Mitochondrion	NC_011033	36
	Plastid	NC_001320	14
Phoenix dactylifera L.	Mitochondrion	NC_016740	36
	Plastid	NC_013991	14
Raphanus sativus L.	Mitochondrion	NC_018551	32
	Plastid	–	–
Ricinus communis L.	Mitochondrion	NC_015141	38
	Plastid	NC_016736	14
Silene latifolia Poir.	Mitochondrion	NC_014487	29
	Plastid	NC_016730	14
Sorghum bicolor (L.) Moench	Mitochondrion	NC_008360	32
	Plastid	NC_008602	12
Spirodela polyrrhiza (L.) Schleid.	Mitochondrion	NC_017840	34
	Plastid	NC_015891	11
Tripsacum dactyloides L.	Mitochondrion	NC_008362	31
	Plastid	–	–
Triticum aestivum L.	Mitochondrion	NC_007579	33
	Plastid	NC_002762	12
Vigna radiata (L.) R. Wilczek	Mitochondrion	NC_015121	31
	Plastid	NC_013843	14
Vitis vinifera L.	Mitochondrion	NC_012119	47
	Plastid	NC_007957	14
Zea mays L.	Mitochondrion	NC_008332	32
	Plastid	NC_001666	12
